# The Bro-Xre toxin-antitoxin modules in *Weissella cibaria*: inducing persister cells to escape tetracycline stress by disrupting metabolism

**DOI:** 10.3389/fmicb.2024.1505841

**Published:** 2024-11-29

**Authors:** Wen-Liang Xiang, Jie Xiong, Han-Yang Wang, Ting Cai, Pei Shi, Qiu-Huan Zhao, Jie Tang, Yi-Min Cai

**Affiliations:** ^1^Food Microbiology Key Laboratory of Sichuan Province, Xihua University, Chengdu, China; ^2^School of Food and Bioengineering, Xihua University, Chengdu, China; ^3^Japan International Research Center for Agricultural Science (JIRCAS), Tsukuba, Japan

**Keywords:** *Weissella cibaria*, Bro-Xre toxin-antitoxin modules, persister cells, tetracycline stress, disrupting metabolism

## Abstract

Toxin-antitoxin (TA) modules are important mediators of persister cell formation in response to environmental stresses. However, the mechanisms through which persistence is controlled remain poorly understood. *Weissella cibaria*, a novel probiotic, can enter a persistent state upon exposure to tetracycline stress. This study found that the Bro-Xre TA modules of *W. cibaria* function as typical tetracycline regulators. The Bro-Xre TA modules were activated when exposed to tetracycline stress, and the released toxin Bro acted on various cellular metabolic processes, including energy, amino acid, and nucleotide metabolism. Among them, the genes related to intracellular energy pathways, such as PTS, EMP, HMP, TCA, and oxidative phosphorylation, were downregulated, leading to reduced ATP synthesis and proton motive force. This metabolic disruption resulted in cells adopting a persistent phenotype, characterized by an increase in cell length in *W. cibaria*. Additionally, the frequency of persister cells increased under tetracycline stress. These results provide a novel perspective for understanding the mechanism by which TA modules induce persistence in probiotics, allowing them to evade antibiotic stress through metabolic disruption.

## 1 Introduction

Antibiotic residues in agricultural environments have caused increasing concern worldwide in recent years. They have potentially negative ecological impacts on the agricultural system, create safety risks to edible crops, and ultimately affect human health. More importantly, when antibiotic residues are absorbed and accumulated by edible plants, they can have a negative impact on subsequent food processing, especially fermentation (Ben et al., 2022). *Weissella cibaria,* a novel probiotic and heterolactic bacterium, is commonly found in various spontaneously fermented foods, which can produce a mild, pleasant aromatic taste, and is an important strain for vegetable fermentation (Liu et al., 2020; Xiang et al., 2020). Currently, accumulating evidence indicates that it can withstand various fermentation-related stresses, including antibiotic residues in raw materials, high acidity, and nutrient starvation caused by fermentation (Gao et al., 2021; Cai et al., 2022). Among them, the problem of antibiotic residues in raw materials has attracted significant attention. However, the escape mechanism of the fermentation strain during antibiotic stress is not yet fully understood.

Typically, bacteria have three survival strategies to escape antibiotic stress: resistance, tolerance, and persistence (Brauner et al., 2016). Of them, the innate persistence of bacteria has attracted more attention because of their ability to regenerate after the cessation of antibiotic stress, regardless of whether bacteria possess antibiotic resistance genes, which was widely considered to be an important reason for the persistent recurrence of bacterial contamination in the food industry (Harms et al., 2018; Kaplan et al., 2021). Under normal conditions, the persistent cells are also present stochastically at a basal rate. However, the regularly growing bacteria rapidly differentiate into persistent cells at a higher rate when stimulated by environmental threats, particularly sub-lethal concentrations of antibiotics (Harms et al., 2018). Many studies have shown that bacterial TA modules are stress response elements that play an important role in mediating bacterial persistence to escape various adverse environments (Hasenoehrl et al., 2019; Huang et al., 2020). They encode two components: a stable toxin protein that inhibits cell growth and a labile antitoxin (either RNA or protein) that regulates toxin activity. Persistence is induced when the toxin protein exceeds a certain threshold in the bacterial cell. These TA modules disrupt the metabolic balance mainly by inhibiting DNA replication, gene transcription, protein synthesis, and ATP production in cells (Wilmaerts et al., 2019a,b), resulting in a reduction in overall cellular metabolism and persistence. The Bro-Xre TA modules in *W. cibaria*, but these responses to antibiotic stress in cells have not yet been studied (Makarova et al., 2009). Interestingly, when we examined the effects of different antibiotic residues, it was found that the Bro-Xre modules of *W. cibaria* responded positively to tetracycline stress, with the greatest response magnitude. However, it was not clear from the data whether the Bro-Xre modules mediated persistence by disrupting the cellular metabolism of *W. cibaria* to escape tetracycline stress.

Therefore, the present study predicted the structure of Bro-Xre modules through bioinformatics analysis in *W. cibaria* 018 and investigated the adverse effects of Bro on basic metabolic processes. Meanwhile, the specific mechanism by which the Bro-Xre modules of *W. cibaria* escape tetracycline stress by mediating persistence through transcriptomics is to be elucidated. The results provide a certain theoretical basis for understanding the mechanism of the Bro-Xre modules that induce the persistence of *W. cibaria* to survive in adverse environments.

## 2 Materials and methods

### 2.1 Bacterial strains and culture conditions

*W. cibaria* 018 (CGMCC 1.19376) was isolated from traditional Sichuan pickles and submitted to the China General Microbiological Culture Collection Center (Beijing, China). The *Escherichia coli* expression strain was the BL21(DE3). All strains were grown in De Man, Rogosa, and Sharpe (MRS) medium or Luria-Bertani medium (LB) at 37°C. Strains were activated in MRS or LB at 37°C for 16 h before experiments.

### 2.2 The effect of tetracycline on the growth of *Weissella cibaria* 018

*Weissella cibaria* 018 was incubated in MRS broth at 37°C until it reached 10^7^ CFU/mL, then 2% of this culture was inoculated into fresh MRS medium with a final concentration of half the minimal inhibitory concentration (MIC) of tetracycline (16 μg/mL) and incubated again at 37°C. Subsequently, bacterial cells were harvested after 0, 1, 3, 5, and 7 h by centrifugation at 4,000 g for 1 min and washed twice with sterile phosphate-buffered saline (PBS) (pH 7.4). After the cell pellets were resuspended and serially diluted 10-fold with PBS, the diluted cells were plated onto an MRS agar plate and counted after 24 h of incubation at 37°C. Treatment without tetracycline was used as the control.

### 2.3 Determination of the cell membrane potential

The cell membrane potential was determined using a fluorescence method with bis (1,3-dibutyl barbituric acid) trimethine oxonol (DiBAC4(3)) (Beijing Solarbio Science & Technology Co., Ltd). The DiBAC4(3) was dissolved in dimethyl sulfoxide to a concentration of 1 mg/mL as a master mix and stored at −20°C. A 1 mL culture of *W. cibaria* 018 treated with tetracycline (16 μg/mL) or *E. coli* BL21 treated with 2.0 g/L arabinose (induced expression of Bro protein) for 0, 1, 3, 5, and 7 h at 37°C was diluted to a bacterial concentration of 10^6^ CFU/mL with sterile PBS containing 10 μg/mL DiBAC4(3), then added to a 96-well plate and incubated for 15 min at room temperature. The fluorescence intensity (MFI) was measured using a Tecan Infinite M1000 Pro reader (Tecan Group, Mannedorf, Switzerland) at 490 nm excitation and 540 nm emission wavelengths. Treatment without tetracycline was used as the control.

### 2.4 Determination of ATP content

The ATP content was determined using an ATP assay kit (Beijing Solarbio Science & Technology Co., Ltd). Briefly, the culture of *W. cibaria* 018 treated with tetracycline or *E. coli* BL21 treated with 2.0 g/L arabinose for 0, 1, 3, 5, and 7 h at 37°C was diluted to a bacterial concentration of 10^6^ CFU/mL with sterile PBS.

Then, the cell dilution (0.5 mL) was mixed thoroughly with lysed fluid (0.5 mL) from the ATP assay kit and placed in an ice bath, subjected to 2 s ultrasound and 1 s intervals for 1 min at 200 W power, followed by centrifugation at 4°C, 10,000 × g for 10 min. The supernatant was collected and mixed thoroughly with 500 μL chloroform at 0°C and then centrifuged at 4°C at 10,000 g for 3 min. The supernatant was collected again and centrifuged twice at 4°C, 10,000 × g for 3 min. The supernatant and ATP standard solution were, respectively, mixed thoroughly with 4°C reagent I and working solution in a volume ratio of 20:128:52, and then immediately measured the absorbance (A1_sample_ and A1_standard_) at 340 nm. The absorbance (A2_sample_ and A2_standard_) was determined again after a rest period of 3 min at 25°C. The ATP content of the bacterial cells was analyzed as follows:

$ATP\;\mathrm{content}=0.125\times \left(\begin{array}{l} A2_{\mathrm{sample}}-\\ A1_{\mathrm{sample}} \end{array}\right)÷\left(\begin{array}{l} A2_{\mathrm{standard}}-\\ A1_{\mathrm{standard}} \end{array}\right)$

### 2.5 Detection of the expression level of Bro-Xre modules in *Weissella cibaria* 018

Total RNA from *W. cibaria* 018 treated with antibiotics in MRS broth for 1 h at 37°C was used as a template for quantitative real-time fluorescence polymerase chain reaction (RT-qPCR) of the Bro-Xre modules. RT-qPCR was performed with TB Green^®^ Premix Ex Taq^™^ II (Takara, Shiga, Japan) and quantified using qTOWER2.0 (Jena, Germany). The RT-qPCR primers used were the *bro*-F (5’-CCAGACAGCCTACGACATTAC-3′) and bro-R (5’-GAGATTGTTTCCAGTCCCTTG-3′) of the *bro* gene and *xre*-F (5’-ATTAAAACAAAAGGGCGTAC-3′) and *xre*-R (5’-TCACTTTGC CTGAATGGA-3′) of the *xre* gene. The reference gene was 16S rRNA (16S rRNA-F: 5’-CGCACAAGCGGTGGAGCAT-3′; 16S rRNA-R: 5’-AACCCAACATCTCACGACAC GA-3′). The reactions were incubated at 95°C for 5 min, followed by 40 cycles at 95°C for 1 min, 55°C for 1 min, and 72°C for 2 min. Relative expression of the Bro-Xre modules was calculated using the 2^−(ΔΔCt)^ method.

### 2.6 The effect of the Bro-Xre modules on *Escherichia coli* BL21

The bro and xre genes were ligated into the plasmids pBAD43 and pET28a to generate recombinant pBAD43-bro and pET28a-xre, respectively. Subsequently, pBAD43-bro and pET28a-xre were co-transformed into *E. coli* BL21 to generate *E. coli*/pBAD43-bro+pET28a-xre (Shen et al., 2016). The *E. coli*/pBAD43-bro+pET28a-xre was inoculated with kanamycin (50 μg/mL) and spectinomycin (100 μg/mL) in LB broth medium. After incubation at 37°C for 2 h, the culture was divided into four parts and incubated again with shaking at 150 × g and 37°C for 10 h. One was used as a control without an inducer. The others were, respectively, used to induce the expression of Xre with 0.1 mmol/L isopropyl *β*-D-1-thiogalactopyranoside (IPTG), the expression of Bro with 2.0 g/L arabinose, and the co-expression of Bro and Xre with 0.1 mmol/L IPTG and 2.0 g/L arabinose. The growth of recombinant *E. coli*/pBAD43-bro+pET28a-xre was monitored hourly at 600 nm using a Tecan Infinite M1000 Pro reader (Tecan Group, Mannedorf, Switzerland).

### 2.7 Determination of persistent cells

Persister cells were detected using the biphasic bactericidal curve method (Harms et al., 2017). Briefly, after the culture of *W. cibaria* 018 was treated with tetracycline (16 μg/mL) in MRS broth for 0, 1, 3, 5, and 7 h at 37°C, respectively, the 0.5 mL treated culture was inoculated into 50 mL MRS broth containing 1.6 mg/mL tetracycline (minimum bactericidal concentration, MBC) and incubated for 24 h at 37°C. Then, 1 mL of incubation solution was harvested by centrifugation at 10,000 × g for 1 min and washed twice with PBS. After the cell pellets were resuspended and serially diluted 10-fold with PBS, the diluted cells were inoculated on MRS agar plates and counted after incubation for 24 h at 37°C. The frequency of persistent cells was expressed as the ratio of viable bacteria in the treated and untreated groups relative to that of tetracycline. The cell morphology of *E. coli* BL21 and *W. cibaria* 018 under tetracycline stress was observed by scanning electron microscopy (SEM) according to the method recommended by Cai et al. (2022).

### 2.8 Isolation of DNA and total RNA, cDNA generation, and co-transcription validation

Total RNA was obtained from *W. cibaria* 018 using the Steadypure Universal RNA Extraction Kit (Accurate Biology, Hunan, China), and RNA quality was assessed using the NanoDrop 2000 (NanoDrop Technologies Inc., Wilmington, DE, United States). The complementary DNA (cDNA) was synthesized using the Evo M-MLV RT Mix Kit with gDNA clean for qPCR Ver. 2 (Accurate Biology, Hunan, China). Genomic DNA was isolated using the Bacterial DNA Isolation Kit (Foregene, Chengdu, China). cDNA was used as a template for PCR amplification, and genomic DNA was used simultaneously as a positive control. PCR was carried out with the primers *xre*-R and *xre*-F for the *xre* gene, *bro*-R and *bro*-F for the *bro* gene, and *xre*-F and *bro*-R for the Bro-Xre modules. Primers *xre*-F/*bro*-R were annealed to the 5′ end of *xre* and the 3′ end of the *bro* coding region, respectively, and gel electrophoresis was used to analyze the PCR products amplified with genomic gDNA and cDNA.

### 2.9 RNA preparation, library construction, and sequence analysis

Total RNA was isolated from *W. cibaria* 018 after treatment with tetracycline in MRS broth or *E. coli* BL21 treated with 2.0 g/L arabinose in LB broth for 1 h at 37°C using the RNAprep pure cell/Bacteria kit (TransGen Biotech, Beijing, China). The treatment without tetracycline or IPTG was the control. RNA quality was evaluated by NanoDrop 2000 (NanoDrop Technologies Inc., Wilmington, DE, United States). The cDNA libraries were synthesized using the PrimeScript^™^RT reagent Kit with gDNA Eraser (Takara, Shiga, Japan), and their length distribution was monitored using a DNA high-sensitivity reagent kit on a Perkin-Elmer Lab chip (Perkin-Elmer, Waltham, MA). All samples were subjected to an indexed paired-end sequencing run of 2 × 51 cycles using an Illumina HiSeq 2000 system (Illumina, San Diego, CA; 16 samples/lane). Raw reads were mapped to the *W. cibaria* 018 using bowtie2 with default parameters. Quantitative analysis of the gene was performed using feature counts and genome annotations from the GenBank file. The differential expression between treatment and control groups was calculated by DESeq 2. A gene with a log2 (fold change) > 1 was defined as a differentially expressed gene (DEG). Subsequently, the DEGs were further carried out for enrichment analysis and functional annotation in the Gene Ontology Resource^1^ and Kyoto Encyclopedia of Genes and Genomes Pathway.^2^

### 2.10 Bioinformatics analysis

The −10 site, −35 site, and termination sites of the promoter on the gene sequence were identified using the online tools BPROM and FindTerm on the Softberry platform.^3^ ClustalX and PSIPRED^4^ were used to determine the primary and secondary structures of bro and xre, respectively. Jalview was then used to visualize the results. The three-dimensional structures of bro and xre were predicted by SWISS-MODEL^5^ based on homology modeling methods, and the results were evaluated by PROCHECK. GRAMM-X^6^ was used to simulate the Bro and Xre models. The HDOCK Server was used to generate a bound DNA model.^7^ Interface areas were calculated using PISA.^8^ The structures were visualized using the PyMOL program.

### 2.11 Statistical analysis

All experiments were performed in triplicate for each sample, and data are presented as the mean ± standard deviation (< 10%). Analysis of significance was performed by analysis of variance (ANOVA) with Tukey’s *post hoc* test in SPSS software, and a *p*-value of <0.05 was considered significant. Graphs were created using GraphPad Prism 9.0 (GraphPad Software, San Diego, CA, United States) and the R software.

## 3 Results

### 3.1 Bro-Xre modules were the typical TA tetracycline stress regulator

Bacterial TA modules played an important role in response to various environmental stresses, including antibiotics, acidity, and nutritional starvation (Zhu et al., 2024; Cai et al., 2022).

**Figure 1:**
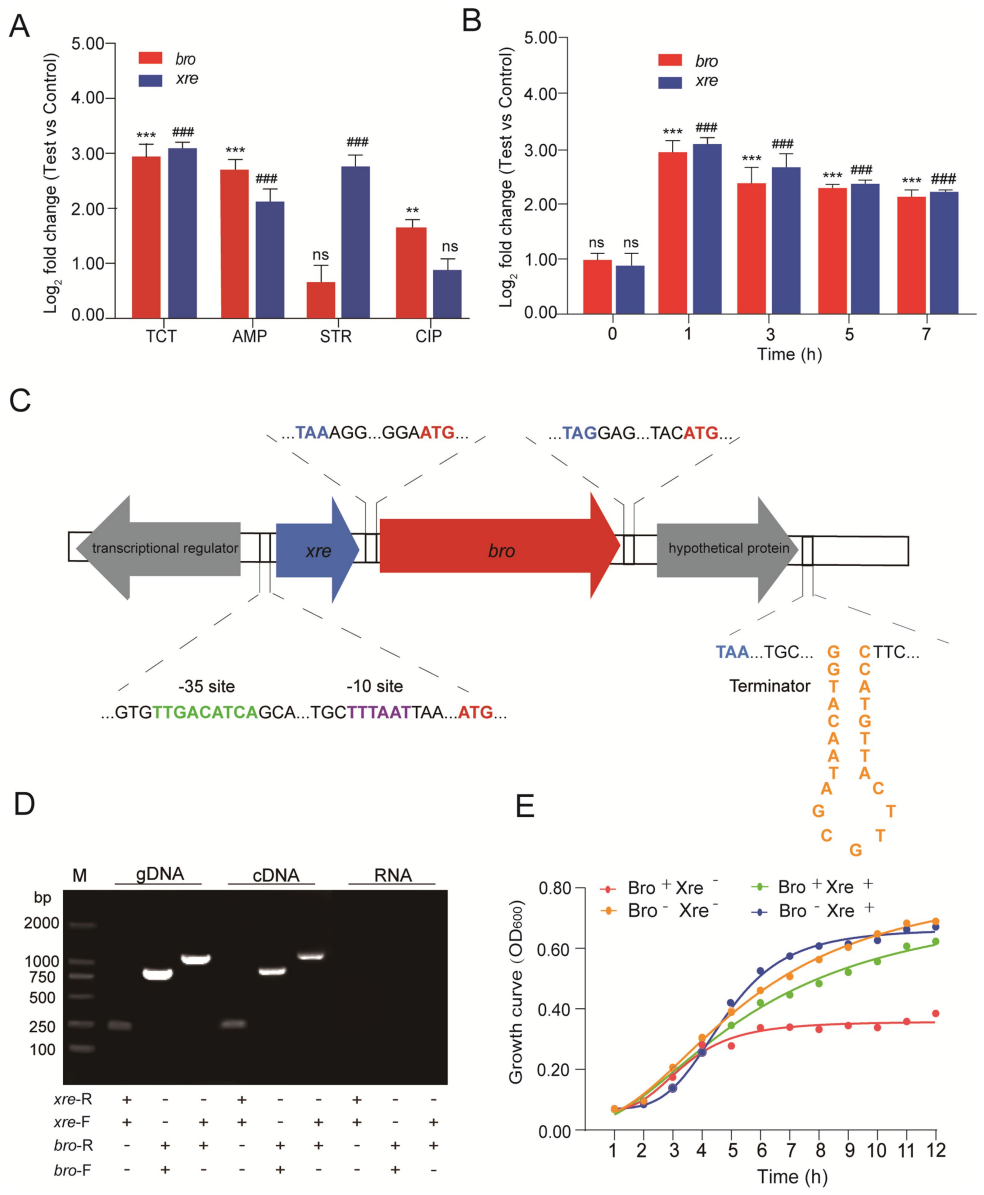
The Bro-Xre modules were the typical TA tetracycline regulator. **(A)** Relative quantification of *bro* and *xre* genes under different antibiotic stresses. The data was determined after being treated with antibiotics at 1 h. The group without antibiotics was used as control, tetracycline: TCT, ampicillin: AMP, streptomycin: STR, and ciprofloxacin: CIP. **(B)** Relative quantification of *bro* and *xre* genes at different times under tetracycline stress. The group without antibiotics was used as a control. **(C)** Operon structure of Bro-Xre in *W. cibaria* 018. **(D)** Validation of co-transcription. Genomic DNA (gDNA) was used as a positive control, and total RNA (RNA) was used as a negative control. M: Marker 2000, gDNA was obtained by genomic DNA, and cDNA was obtained by Reverse transcription DNA. **(E)** Growth curve of *E. coli*/pBAD43-bro + pET28a-xre. After 2 h of incubation for *E. coli*/pBAD43-bro +pET28a-xre, the culture was divided into four tubes: Bro+ Xre−: Induce toxin expression, Bro+ Xre+: Induce co-expression of toxin and antitoxin, Bro− Xre+: Induce antitoxin expression, Bro-Xre-: Negative control. Data were analyzed using by ANOVA with Tukey’s *post hoc* test. The ns: *p* > 0.05; # and *: *p* < 0.05; ## and **: *p* < 0.01; ### and ***: *p* < 0.001.

For several of the antibiotics tested, we found that the magnitude of the response was greater under tetracycline stress (Figure 1A). After treatment with tetracycline for 1 h, the transcription of *bro* and *xre* genes was upregulated by 7.01 and 7.88 folds, respectively. In addition, the transcription levels were upregulated by 3.18–7.26 folds and 4.11–7.41 folds during 3–7 h, respectively (Figure 1B). BPROM (Bacterial sigma70 Promoter Prediction Program) and FindTerm software were used to analyze the complete genome sequence of *W. cibaria* 018. The results found that the *xre* antitoxin gene was located 23 bp upstream of the *bro* toxin gene. The Sigma70 promoter, including sites −35 (TTGACATCA) and − 10 (TTTAAT), was located 31 bp upstream of the ATG start codon of the xre gene. There was a transcriptional regulator on the upstream antisense chain of the *xre* gene and a putative protein downstream of the *bro* gene. A promoter was found upstream of the *xre* gene, but a transcriptional terminator was downstream of the putative protein found, not downstream of the *bro* gene (Figure 1C). Therefore, the Bro-Xre modules may be similar to the paaA2-parE2 system on the prophage of *Escherichia coli* O157:H7 (Jurenas et al., 2021), in that the postulated protein behind Bro is involved in the self-regulation of its modules.

Co-localization and co-transcription in an operon are common characteristics of TA modules (Zhou et al., 2021). Our results showed that the expected PCR products were found in the cDNA and gDNA templates (Figure 1D), proving that *xre* and *bro* were expressed in a bicistronic operon. When *W. cibaria* 018 was exposed to tetracycline stress, the co-transcription of Bro-Xre modules was activated (Figure 1D), while Xre was degraded by the protease.

Consequently, free Bro exhibited cytotoxicity to *W. cibaria* 018. The growth of *E. coli*/pBAD43-bro+pET28a-xre was significantly inhibited after Bro induction, and the growth of *E. coli*/pBAD43-bro+pET28a-xre was restored when Bro and Xre were induced simultaneously (Figure 1E). Additionally, antitoxin Xre had a detoxifying effect. Therefore, the Bro-Xre modules of *W. cibaria* 018 were the typical TA tetracycline stress regulators.

### 3.2 Analysis of Bro-Xre modules structure in *Weissella cibaria*

**Figure 2:**
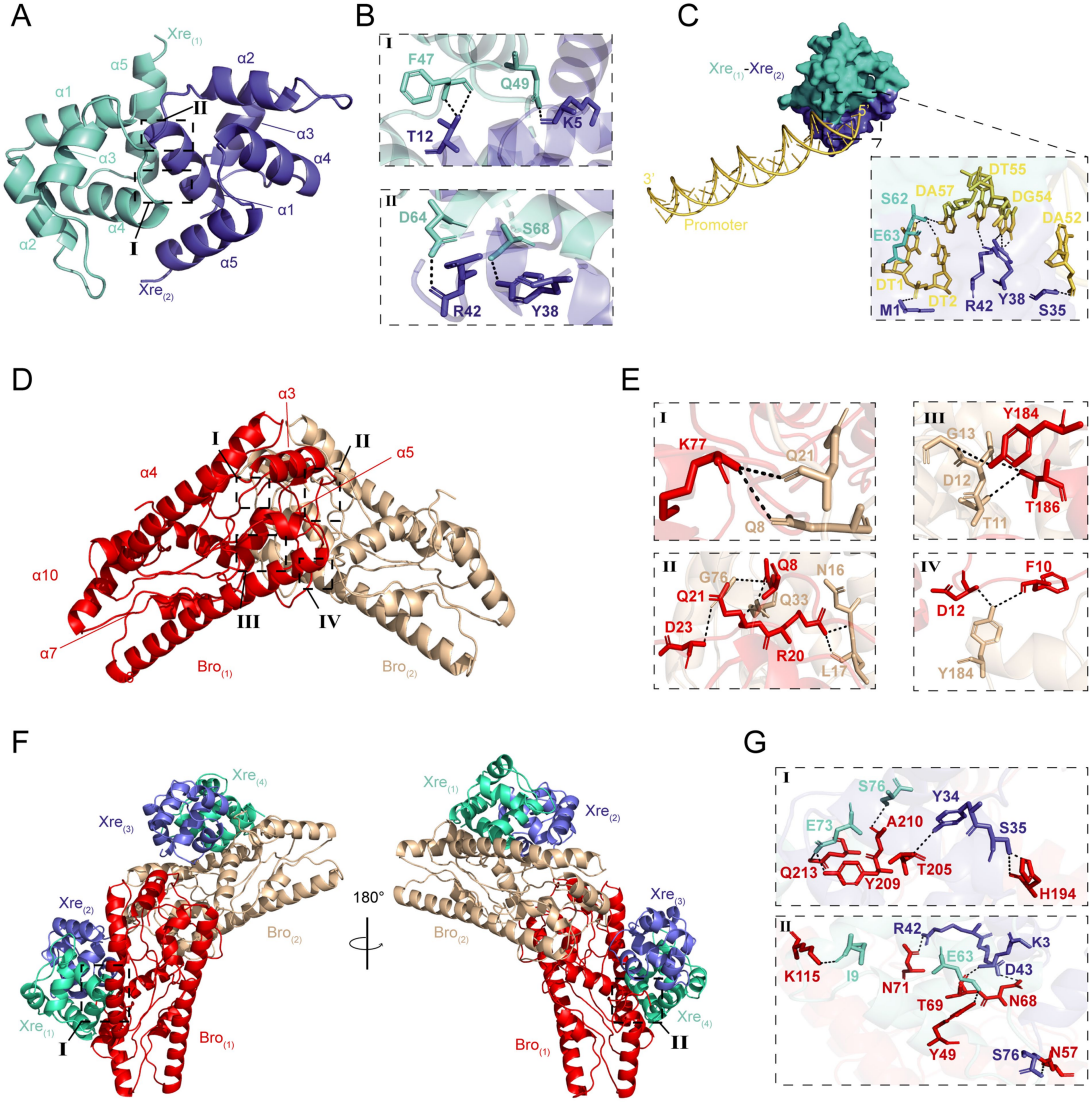
The predicted results on the structure of Bro-Xre models. **(A)** The homodimers of Xre_(1)_-Xre_(2)_, the *α* refer to α-helix. **(B)** Residues involved in hydrogen bond formation in the Xre_(1)_-Xre_(2)_ dimers. **(C)** Xre_(1)_-Xre_(2)_-linked promoter trimer structure created by the HDOCK webserver. The yellow double helix structure represented promoter −35 to −10 sites, and the dashed box contains the residues involved in the formation of hydrogen bonds. **(D)** The homodimers of Bro_(1)_-Bro_(2)_. **(E)** Residues involved in hydrogen bond formation in the Bro_(1)_-Bro_(2)_ dimers. **(F)** Overview of the (Xre)_2_-(Bro)_2_-(Xre)_2_ hexamer in two perpendicular orientations with the Bro toxin molecules in red/brown and the Xre antitoxin molecules in green/purple cartoons. **(G)** Residues involved in hydrogen bond formation in the (Xre)_2_-(Bro)_2_-(Xre)_2_ hexamer.

The Bro and Xre in *W. cibaria* 018 consist of 262 and 76 amino acids with molecular weights of 30.5 and 8.7 kDa, respectively, and they have 100 and 25% similarity with those from *Lactiplantibacillus plantarum* and *Lactobacillus parabuchneri*, respectively (Supplementary Figure S1). We simulated the three-dimensional structures of Bro and Xre using homologous modeling, where Bro consisted of β1α1α2β2α3 (Supplementary Figures S2A,C), while Xre consisted of α1α2α3α4α5 (Supplementary Figures S2B,D). In the context of the Bro-Xre complex, our results indicated that two Xre molecules and two Bro molecules formed Xre_(1)_-Xre_(2)_ (Figures 2A,B; Supplementary Tables S1–S3) and Bro_(1)_-Bro_(2)_ homodimers (Figures 2D,E; Supplementary Tables S6, S7), respectively. Each Bro molecule within the central toxin dimer then associates with a V-shaped homodimer of Xre antitoxin molecules in a 1:2 toxin-to-antitoxin stoichiometry, forming a hexameric complex (Xre)2-(Bro)2-(Xre)2 (Figures 2F,G; Supplementary Tables S8–S13), which aligns with previous findings (Skjerning et al., 2019). The antitoxin has an Xre domain with 5–66 amino acid residues, which can directly bind to the promoter DNA and participate in transcription control as a regulatory factor, thereby achieving TA module self-regulation (Song et al., 2022). The promoter modules for Xre binding created by the HDOCK Server are shown in Figure 2C. The confidence value and docking score of the model are 0.84 and − 231.41 kcal/mol, respectively, showing that the docking was positive (Supplementary Tables S4, S5). Under normal conditions, the toxin of the TA modules combined with homologous antitoxin produces a stable molecule that inhibits toxin function (Chan et al., 2016). The majority of antitoxins are destroyed by Lon or Clp proteases under environmental stresses, such as antibiotic therapy, acid, and nutrition deprivation, activating the TA modules (Muthuramalingam et al., 2016; Harms et al., 2018).

### 3.3 Effect of the toxin protein bro on energy metabolism

**Figure 3:**
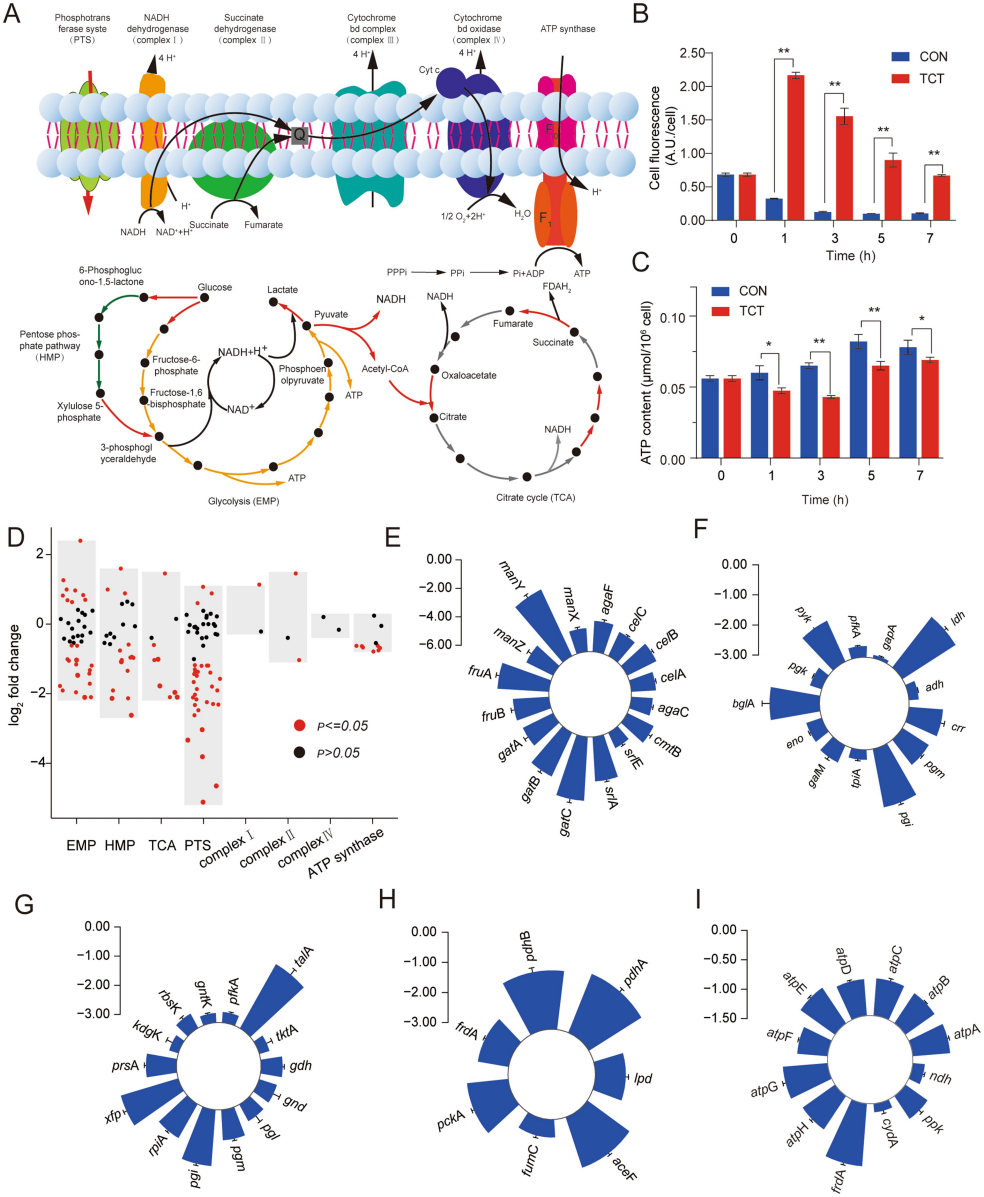
Changes of central carbon metabolism and oxidative phosphorylation gene expression in *W. cibaria* 018 induced by tetracycline stress. **(A)** Diagram of central carbon metabolism and oxidative phosphorylation. **(B)** The change of cell membrane potential in *W. cibaria* 018, the sample without antibiotics, was used as a control. **(C)** The change of ATP content in *W. cibaria* 018, the sample without antibiotics, was used as a control. **(D)** Effect of tetracycline stress on gene expression of *W. cibaria* 018. **(E)** PTS. **(F)** EMP. **(G)** HMP. **(H)** TCA. **(I)** ATP synthases.

*W. cibaria* 018 was a facultatively anaerobic parthenogenic bacterium that derives its energy primarily from heterolactic fermentation or oxidative phosphorylation and has a complete electron transport chain and ATP synthase. The phosphotransferase system (PTS) was the bacteria’s primary mechanism for carbohydrate uptake. Under anaerobic conditions, glucose 6-phosphate is metabolized via the hexose monophosphate pathway (HMP) and the Embden-Meyerhof-Parnas pathway (EMP) to produce a molecule of lactic acid and ethanol. Under aerobic conditions, glucose 6-phosphate distributes throughout the EMP to produce two molecules of NADH and pyruvate. Pyruvate undergoes a decarboxylation reaction and TCA to generate 4 molecules of NADH and one molecule of FADH_2_. These NADH and FADH_2_ undergo oxidative phosphorylation, form a proton force, and synthesize a large amount of ATP (Figure 3A).

Persisters are typically described as dormant cells that survive antibiotic treatment and have low cellular energy metabolism. Reduced cellular energy levels may be the result of inhibition of ATP production or leakage of ATP from the cell (Wilmaerts et al., 2019a,b). Manuse et al. (2021) found that cells with low ATP levels exhibited extreme tolerance to current antibiotics. We measured the changes in ATP concentration and cell membrane potential that are closely related to the formation of persister cells in *W. cibaria* 018 under tetracycline stress. In the CON group, the fluorescence intensity at 0 h was the strongest (0.75 A.U./Cell) and gradually decreased with the culture time, which may be due to the reason that the cells at 0 h were still in the adaptation stage to the environment, and the absorption of the nutrient from the environment was weaker than in the late growth stage. During the entire incubation period, the fluorescence intensity of DiBAC4(3) in the tetracycline (TCT) group, which was negatively correlated with the cell membrane potential (Taggar et al., 2021), was significantly higher than that of the CON group (*p* < 0.05), and after 3 h the fluorescence intensity of the experimental group was 12 times higher than that of CON (Figure 3B). In CON, the ATP content of *W. cibaria* 018 increased gradually. In contrast, the ATP content in the TCT group initially decreased and reached a minimum value after 3 h (0.04 μmol/10^6^ cells), then slowly increased again and reached 0.69 μmol/10^6^ cells after 7 h (Figure 3C). Similar results of ATP and cell membrane potential were found in *E. coli* BL21, which induced Bro protein (Supplementary Figures S3A,B), which suggested the Bro-Xre modules were activated under tetracycline stress, the Bro protein was released and acted on the cell process, changed the cell membrane potential, and changed the ATP content.

Overall, most genes involved in energy metabolism were downregulated in *W. cibaria* 018 (Figure 3D). In the PTS pathway, we detected that after *W. cibaria* 018 was treated with tetracycline, 53.7% of the PTS-related genes, such as *srl*A, *gat*B, *fru*A, *gat*C, and *man*Y, were significantly downregulated about eight times (Figure 3E). Furthermore, in the EMP pathway, the rate-limiting genes *pgm* and *pyk* were downregulated by 2.05 and 2.62 folds, respectively (Figure 3F), while the phosphoketolase gene *xfp* was downregulated by 4.39 folds (Figure 3G), resulting in the absorption of glucose, glycolysis, and the metabolic activity of heterolactic acid being decreased.

Pyruvate can only enter the TCA cycle after being converted to acetyl-CoA by dehydrogenase. However, the genes encoding dehydrogenases (*ace*F, *pdh*A, and *pdh*B) were downregulated by 3.43-fold, 4.29-fold, and 3.90-fold, respectively (Figure 3H), leading to decreased TCA cycle activity. In addition, the transcription level of *ndh*-encoded NADH dehydrogenase was upregulated by 2.44-fold, while genes encoding ATP synthases (*atp*A, *atp*B, *atp*C, *atp*D, *atp*E, *atp*G, and *atp*H) were significantly downregulated by less than 2-fold (Figure 3I). The majority of the genes showed similar downgrades in energy metabolism in *E. coli* BL21 when induced by the Bro protein (Supplementary Figure S3C). These results demonstrated that the Bro protein could reduce *W. cibaria* 018 energy metabolism.

### 3.4 Effect of the toxin protein bro on amino acid and nucleotide metabolism

**Figure 4:**
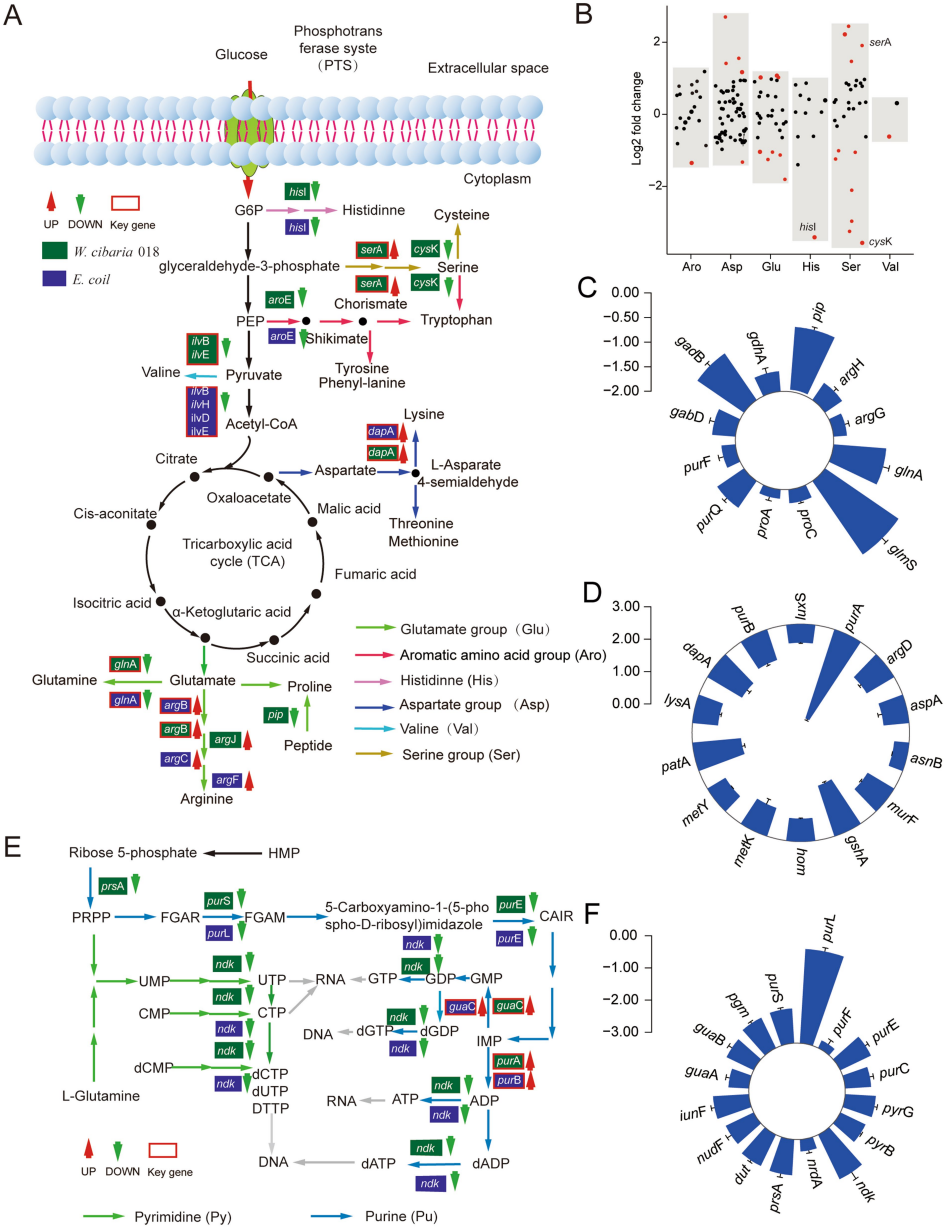
Expression changes of amino acid and nucleotide synthesis genes in *W. cibaria* 018 induced by tetracycline stress. **(A)** Diagram of amino acid synthesis. **(B)** Effect of tetracycline on amino acid synthesis gene expression of *W. cibaria* 018. **(C)** Glutamate metabolism. **(D)** Aspartate metabolism. **(E)** Diagram of nucleotide synthesis. **(F)** The changes of nucleotide synthesis gene expression.

The amino acids are an essential component of proteins and an important source of nitrogen and sulfur and play a central role in the entire metabolism of the organism (Song et al., 2021). After *W. cibaria* 018 was treated with tetracycline, the *ser*A gene involved in the synthesis of serine precursors (glycerate) was upregulated, while the *cys*K genes, which converts to cysteine, were downregulated, resulting in intracellular accumulation of serine (Figures 4A,B). Similarly, the genes *arg*J and *arg*B, which were involved in converting glutamate to arginine, and the *arg*F gene, which was involved in further converting ornithine to arginine, were upregulated (Figure 4A). While the *gln*A and *gad*B genes were involved in the conversion of glutamine and *γ*-aminobutyric acid, respectively, as well as the downregulation of the *glm*S gene, which was involved in deamination in carbon metabolism, promoted the synthesis of arginine in the cells (Figure 4C). Among other amino acids, the expression of histidine synthesis genes (*his*I) was suppressed (Figure 4B), while conversion of aspartate to lysine (*dap*A, *pat*A) and glycine metabolism (GCSH) related gene *gcv*H was upregulated (Figure 4D).

The end product of the nucleotide metabolic pathway is the raw material for nucleic acid synthesis, and other substances produced in the metabolic process also have important biological functions. Under tetracycline stress, the expression levels of 12 genes in *W. cibaria* 018 were changed, and the expression levels of *pur*A and *gua*C regulated by autogenic products were significantly increased (Figure 4E). The nucleotide metabolic pathway of *W. cibaria* 018 was significantly downregulated in related genes (Figures 4E,F), potentially inhibiting the formation of *W. cibaria* 018 ribonucleotides. Overall, the decrease in the production of ATP, AMP, cAMP, and cGMP affects the overall metabolism of cells, resulting in slow cell growth. The majority of the genes showed similar downgrades in amino acid and nucleotide metabolism in *E. coli* BL21 with inducing Bro toxin proteins (Figure 4A; Supplementary Figures S3D,E).

### 3.5 The bro protein escapes tetracycline stress by forming the persister cells

**Figure 5:**
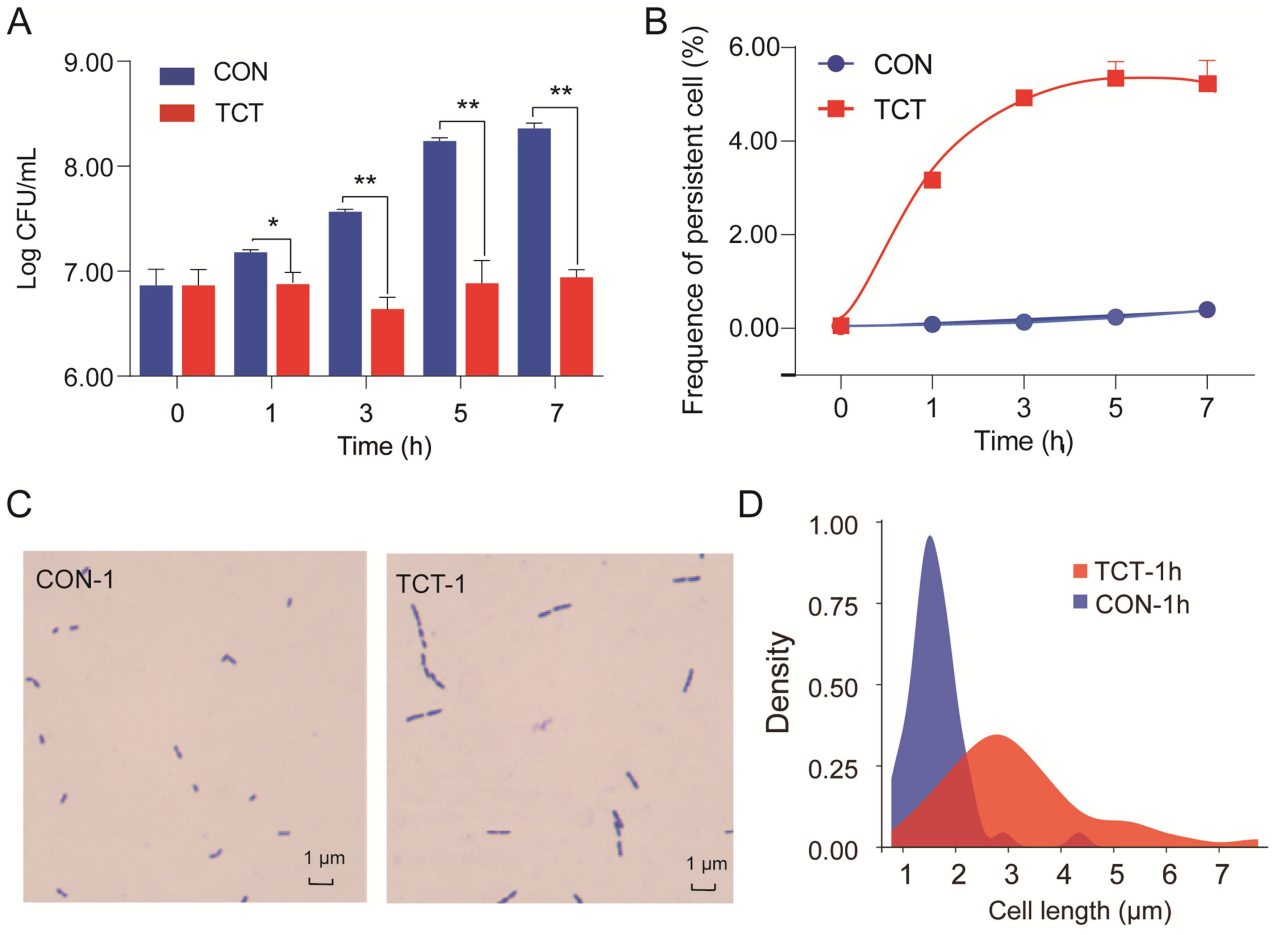
Bro protein promotes the formation of persister cells. **(A)** Effects of tetracycline stress on the growth of *W. cibaria* 018: strain count was performed using the agar plate after 24 h of incubation. **(B)** The frequency of persister cells of *W. cibaria* 018 under tetracycline stress. It was performed by a biphasic extinction curve. **(C)** The effect of Bro of *W. cibaria* 018 on cell morphology. **(D)** Effect of tetracycline stress on the cell length. Data were analyzed using ANOVA with Tukey’s *post hoc* test. The ns: *p* > 0.05; *: *p* < 0.05; **: *p* < 0.01; ***: *p* < 0.001.

Activation of the toxin of bacterial TA modules inhibits bacterial growth and plays an important role in persister formation (Li et al., 2016; Urban-Chmiel et al., 2022). In our study, the number of viable cells decreased from 8.36 log10 CFU/mL to 6.94 log10 CFU/mL when *W. cibaria* 018 was exposed to the tetracycline stress for 7 h compared to the CON group (Figure 5A), and the biphasic extinction curve showed a significant difference in the frequency of persistent cells between the TCT group and the CON group. The TCT group increased rapidly to 3.17% in 1 h and stabilized at 5.30% from 5 h to 7 h compared to the CON group (Figure 5B).

Under tetracycline stress, the Bro-Xre TA modules were activated, and the Bro toxin was released. The growth of filamentous cells may occur during the formation of persister cells (Jones and Uphoff, 2021). We observed an increase in the cell length of *W. cibaria* 018 from the usual 1.1–2.1 μm to 1.8–5.4 μm, even with a maximum length of 7.74 μm (Figures 5C,D). The same filamentous cells were also found, and the frequency of persistent cells increased in recombinant *E. coli* BL21 with inducing Bro toxin protein (Supplementary Figure S4). Toxin expression does not necessarily lead to changes in the morphology of persister cells, but when it does, it usually involves abnormal elongation, filamentation, and even a transition from spiral to round coccoid (Yu et al., 2023; El Mortaji et al., 2020; Zhu et al., 2024). Therefore, the Bro protein has prolonged the persister cells of *W. cibaria* under tetracycline stress through the same mechanism as *E. coli* BL21.

## 4 Discussion

Tetracycline is increasingly used as a broad-spectrum bacteriostatic agent to promote animal growth and prevent animal diseases in animal husbandry (Hu et al., 2021). It plays an important role in the development of animal husbandry, which society also recognized and confirmed. However, the excessive and continuous utilization of tetracycline in recent years has caused residues in food (Zhang et al., 2024; Chen et al., 2019). Once the tetracyclines in this food enter the fermentation system, they influence microbial community succession and flavor formation during the fermentation process (Xiang et al., 2022). *W. cibaria* was better at initiating heterolactic fermentation and producing a mild, pleasantly aromatic flavor during the fermentation. In *W. cibaria*, the response to antibiotic residues was crucial for fermented products’ flavor development and product quality. As a regulatory element of cellular metabolism, the TA modules could alter the normal physiological state of cells to form persistence by releasing toxin proteins to cope with various environmental stresses (Amraei et al., 2020a; Amraei et al., 2020b; Yu et al., 2023). Our presented results revealed the molecular mechanism by which Bro toxin protein induced *W. cibaria* persistence by disrupting crucial metabolism (Figures 3–5), including energy, amino acid, and nucleotide metabolism. These results provided valuable insights into how *W. cibaria* escaped tetracycline stress during fermentation.

Bacterial persistence is a programmed phenotypic transformation with a genetic basis. Currently, it is widely accepted that TA modules play a crucial role in bacterial persistence (Page and Peti, 2016). Our results further supported this consensus. Bro-Xre TA modules were the typical TA tetracycline stress regulator (Figure 1). The structure of Bro-Xre modules from *W. cibaria* 018 was similar to RES-Xre of *Photorhabdus luminescens* (Skjerning et al., 2019). It was demonstrated that the TA complex forms a hexamer, W-shaped assembly on the form (Xre)_2_-(Bro)_2_-(Xre)_2_ (Figure 2F). To our knowledge, this 1:2 toxin: antitoxin stoichiometry was rarely reported in TA modules. In general, the highly dynamic antitoxins in TA modules are composed of two separate domains: an N-terminal DNA-binding domain through which it can negatively regulate its own promoter and a C-terminal toxin-binding domain (Eun et al., 2024; Zorzini et al., 2015). In the present study, we docked the antitoxin dimer (Xre)_2_ with the predicted promoter sequence (Figure 2C). However, the specific mechanism of regulation of the Bro-Xre operon was not known.

The Bro-Xre TA modules were activated when *W. cibaria* 018 was exposed to tetracycline stress during fermentation. Xre antitoxins can be selectively degraded. Subsequently, the free toxin Bro was released and affected various cellular processes, including energy, amino acid, and nucleotide metabolism (Figures 3, 4), which caused the cellular phenotype to change from sensitive to persistent type, and then the frequency of persistent cells increased significantly (Figure 5; Supplementary Figure S4). Energy metabolism is closely related to the formation of persister cells (Kawai et al., 2019). Shan et al. (2017) reported that variation in ATP levels led to persister formation by decreasing the activity of antibiotic targets in *Escherichia coli*. *Staphylococcus aureus* persisters were produced due to a stochastic entrance into the stationary phase and a decrease in intracellular ATP (Conlon et al., 2016). Our results demonstrated the proton motive force and ATP content significantly decreased in *W. cibaria* 018 under tetracycline stress (Figures 3B,C). Similar results were found in *E. coli* BL21, which induced Bro protein (Supplementary Figure S3). Proton motive force and ATP played an important role in antibiotic absorption and the bactericidal activity of antibiotics (Conlon et al., 2016; Deng et al., 2020). Wilmaerts et al. (2019a,b) found that the HokB toxin protein can promote the formation of persister cells by reducing ATP content in cells and reducing proton kinetic potential. In the present study, the toxin protein Bro acted on its target and inhibited the transcription of most transporters in PTS, thereby reducing the absorption of glucose and other carbon sources, thus inhibiting the carbon source metabolism (Figure 3E; Supplementary Figure S3C).

Meanwhile, the toxin protein Bro inhibited the transcripts of key enzymes of EMP and HMP (Figures 3F,G), such as pyruvate kinase, phosphoglucomutase, and phosphoketolase in *W. cibaria* 018. The reduction of key enzymes led to the stagnation of HMP and EMP, resulting in NADH production in TCA. ATP synthesis in the EMP and oxidative phosphorylation processes was further reduced (Figure 3I). Meanwhile, nucleic acid metabolism also showed that the generation of AMP, cAMP, and cGMP was reduced under tetracycline stress, affecting the cell metabolism (Figures 4E,F; Supplementary Figure S3E).

In addition, the toxin protein Bro affected the metabolic level of amino acids (Figure 4A; Supplementary Figure S3D). Among them, Bro protein promoted serine accumulation. The study showed that high intracellular concentrations of serine could activate the bifunctional (p)ppGpp synthetase/hydrolase SpoT, leading to the accumulation of (p)ppGpp (Zhou et al., 2021). Germain et al. (2013) reported that HipA mediated persistence by triggering the synthesis of (p)ppGpp. In the presence of reduced levels of most amino acid synthesis, the toxin protein Bro promotes the production of large amounts of arginine, spermine, and spermidine, thus ensuring the stability of cellular metabolism (Figure 4A). Polyamines (putrescine, spermidine, and spermine) are essential for normal cell growth and can modulate the functions of DNA, nucleotide triphosphates, proteins, and especially RNA (Dever and Ivanov, 2018; Igarashi and Kashiwagi, 2010). The report found that spermidine not only improved the accuracy and rate of protein synthesis and regulated the cell cycle to restore cell growth (Igarashi and Kashiwagi, 2019) but also regulated the SOS response induced by colicin E7 production after DNA damage (Chagneau et al., 2019), helping fight against oxidative stress by directly interacting with free radicals or by altering gene expression to moderate the DNA damage caused by •OH.

## 5 Conclusion

Overall, the study demonstrated that Bro-Xre modules function as typical TA (toxin-antitoxin) tetracycline stress regulators. The Bro-Xre module of *W. cibaria* 018 formed a hexamer structure: (Xre)_2_-(Bro)_2_-(Xre)_2_. Under tetracycline stress, the Bro-Xre modules were activated, releasing the free toxin Bro, which affected various cellular processes, including energy metabolism, amino acid, and nucleotide metabolism. This stress also reduced the expression of key enzyme genes involved in pathways such as EMP, HMP, TCA, and oxidative phosphorylation. As a result, proton motive force and ATP content significantly decreased, leading to the formation of persister cells. These results provide a novel perspective on the escape mechanism of fermentation strains under antibiotic stress. Further research, including gene knockout experiments, is needed for ontology validation.

## Data Availability

The original contributions presented in the study are included in the article/Supplementary material, further inquiries can be directed to the corresponding author.

## Footnotes

^1^http://geneontology.org/

^2^https://www.genome.jp/kegg/

^3^http://www.softberry.com/

^4^http://bioinf.cs.ucl.ac.uk/psipred/

^5^https://swissmodel.expasy.org/

^6^https://gramm.compbio.ku.edu/

^7^http://hdock.phys.hust.edu.cn/

^8^https://www.ebi.ac.uk/msd-srv/prot_int/pistart.html

## References

[ref1] Amraei F. Narimisa N. Kalani B. S. Lohrasbi V. Jazi M. F. (2020b). Persister cells formation and expression of type II toxin-antitoxin system genes in *Brucella melitensis* (16M) and *Brucella abortus* (B19). Iran. J. Pathol. 15, 127–133. doi: 10.30699/ijp.2020.118902.2294, PMID: 32215028 PMC7081757

[ref2] Amraei F. Narimisa N. Kalani S. B. Mohammadzadeh R. Lohrasbi V. Jazi M. F. (2020a). The expression of type II TA system genes following exposure to the sub-inhibitory concentration of gentamicin and acid stress in Brucella spp. Microb. Pathogenesis. 144:104194. doi: 10.1016/j.micpath.2020.104194 32289464

[ref3] Ben Y. J. Hu M. Zhong F. X. Du E. Li Y. Zhang H.. (2022). Human daily dietary intakes of antibiotic residues: dominant sources and health risks. Environ. Res. 212:113387. doi: 10.1016/j.envres.2022.113387 35513060

[ref4] Brauner A. Fridman O. Gefen O. Balaban N. Q. (2016). Distinguishing between resistance, tolerance and persistence to antibiotic treatment. Nat. Rev. Microbiol. 14, 320–330. doi: 10.1038/nrmicro.2016.34, PMID: 27080241

[ref5] Cai T. Zhao Q. Xiang W. Zhu L. Rao Y. Tang J. (2022). HigBA toxin–antitoxin system of *Weissella cibaria* is involved in response to the bile salt stress. J. Sci. Food Agr. 102, 6749–6756. doi: 10.1002/jsfa.12042, PMID: 35633128

[ref6] Chagneau C. V. Garcie C. Bossuet-Greif N. Tronnet S. Brachmann A. O. Piel J.. (2019). The polyamine spermidine modulates the production of the bacterial genotoxin colibactin. mSphere 4, e00414–e00411. doi: 10.1128/msphere.00414-19, PMID: 31578245 PMC6796968

[ref7] Chan W. T. Espinosa M. Yeo C. C. (2016). Keeping the wolves at bay: antitoxins of prokaryotic type II toxin-antitoxin systems. Front. Mol. Biosci. 3:Article 9. doi: 10.3389/fmolb.2016.00009, PMID: 27047942 PMC4803016

[ref8] Chen J. Ying G. G. Deng W. J. (2019). Antibiotic residues in food: extraction, analysis and human health concerns. J. Agr. Food. Chem. 67, 7569–7586. doi: 10.1021/acs.jafc.9b01334, PMID: 31198037

[ref9] Conlon B. P. Rowe S. E. Gandt A. B. Nuxoll A. S. Donegan N. P. Zalis E. A.. (2016). Persister formation in *Staphylococcus aureus* is associated with ATP depletion. Nat. Microbiol. 1:16051. doi: 10.1038/nmicrobiol.2016.51, PMID: 27572649 PMC4932909

[ref10] Deng W. Fu T. W. Zhang Z. Jiang X. Xie J. P. Sun H.. (2020). L-lysine potentiates aminoglycosides against *Acinetobacter baumannii* via regulation of proton motive force and antibiotics uptake. Emerg. Microbes. Infec. 9, 639–650. doi: 10.1080/22221751.2020.1740611, PMID: 32192413 PMC7144275

[ref11] Dever T. E. Ivanov I. P. (2018). Roles of polyamines in translation. J. Biol. Chem. 293, 18719–18729. doi: 10.1074/jbc.tm118.003338, PMID: 30323064 PMC6290161

[ref12] El Mortaji L. Tejada-Arranz A. Rifflet A. Boneca I. G. Pehau-Arnaudet G. Radicella J. P.. (2020). A peptide of a type I toxin-antitoxin system induces *Helicobacter pylori* morphological transformation from spiral shape to coccoids. PNAS 117, 31398–31409. doi: 10.1073/pnas.2016195117, PMID: 33229580 PMC7733810

[ref9001] Eun H. J. Lee S. Y. Lee K. Y. (2024). DNA binding reveals hidden interdomain allostery of a MazE antitoxin from Mycobacterium tuberculosis. Biochem. Bioph. Res. Co. 710:149898. doi: 10.1016/j.bbrc.2024.149898 38598903

[ref13] Gao Y. Q. Niu M. Z. Yu X. H. Bao T. T. Wu Z. W. Zhao X. (2021). Horizontally acquired polysaccharide-synthetic gene cluster from *Weissella cibaria* boosts the probiotic property of Lactiplantibacillus plantarum. Front. Microbiol. 12:692957. doi: 10.3389/fmicb.2021.692957, PMID: 34234766 PMC8256895

[ref14] Germain E. Castro-Roa D. Zenkin N. Gerdes K. (2013). Molecular mechanism of bacterial persistence by HipA. Mol. Cell 52, 248–254. doi: 10.1016/j.molcel.2013.08.045, PMID: 24095282

[ref15] Harms A. Brodersen D. E. Mitarai N. Gerdes K. (2018). Toxins, targets, and triggers: an overview of toxin-antitoxin biology. Mol. Cell 70, 768–784. doi: 10.1016/j.molcel.2018.01.003, PMID: 29398446

[ref16] Harms A. Fino C. Sørensen M. A. Semsey S. Gerdes K. (2017). Prophages and growth dynamics confound experimental results with antibiotic-tolerant persister cells. MBio 8, e01964–e01917. doi: 10.1128/mbio.01964-17, PMID: 29233898 PMC5727415

[ref17] Hasenoehrl E. J. Sajorda R. D. Berney-Meyer L. Johnson S. Tufariello J. M. Fuhrer T.. (2019). Derailing the aspartate pathway of *Mycobacterium tuberculosis* to eradicate persistent infection. Nat. Commun. 10:4215. doi: 10.1038/s41467-019-12224-3, PMID: 31527595 PMC6746716

[ref18] Hu X. L. Zhao Y. Q. Dong J. Y. Liu C. Qi Y. Fang G. Z.. (2021). A strong blue fluorescent nanoprobe based on mg/N co-doped carbon dots coupled with molecularly imprinted polymer for ultrasensitive and highly selective detection of tetracycline in animal-derived foods. Sensor. Actuat. B-Chem. 338:129809. doi: 10.1016/j.snb.2021.129809

[ref19] Huang C. Y. Gonzalez-Lopez C. Henry C. Mijakovic I. Ryan K. R. (2020). hipBA toxin-antitoxin systems mediate persistence in *Caulobacter crescentus*. Sci. Rep. 10:2865. doi: 10.1038/s41598-020-59283-x, PMID: 32071324 PMC7029023

[ref20] Igarashi K. Kashiwagi K. (2010). Modulation of cellular function by polyamines. Int. J. Biochem. Cell Biol. 42, 39–51. doi: 10.1016/j.biocel.2009.07.009 19643201

[ref21] Igarashi K. Kashiwagi K. (2019). The functional role of polyamines in eukaryotic cells. Int. J. Biochem. Cell Biol. 107, 104–115. doi: 10.1016/j.biocel.2018.12.012, PMID: 30578954

[ref22] Jones E. C. Uphoff S. (2021). Single-molecule imaging of LexA degradation in *Escherichia coli* elucidates regulatory mechanisms and heterogeneity of the SOS response. Nat. Microbiol. 6, 981–990. doi: 10.1038/s41564-021-00930-y, PMID: 34183814 PMC7611437

[ref23] Jurenas D. Fraikin N. Goormaghtigh F. de Bruyn P. Vandervelde A. Zedek S.. (2021). Bistable expression of a toxin-antitoxin system located in a cryptic prophage of *Escherichia coli* O157:H7. MBio 12:e0294721. doi: 10.1128/mbio.02947-21, PMID: 34844426 PMC8630535

[ref24] Kaplan Y. Reich S. Oster E. Maoz S. Levin-Reisman I. Ronin I.. (2021). Observation of universal ageing dynamics in antibiotic persistence. Nature 600, 290–294. doi: 10.1038/s41586-021-04114-w, PMID: 34789881

[ref25] Kawai Y. Mercier R. Mickiewicz K. Serafini A. de Carvalho L. P. S. Errington J. (2019). Crucial role for central carbon metabolism in the bacterial L-form switch and killing by beta-lactam antibiotics. Nat. Microbiol. 4, 1716–1726. doi: 10.1038/s41564-019-0497-3, PMID: 31285586 PMC6755032

[ref26] Li M. Long Y. Q. Liu Y. Liu Y. Chen R. H. Shi J.. (2016). HigB of *Pseudomonas aeruginosa* enhances killing of phagocytes by up-regulating the type III secretion system in ciprofloxacinf induced persister cells. Front. Cell. Infect. Microbiol. 6:125. doi: 10.3389/fcimb.2016.00125, PMID: 27790409 PMC5064212

[ref27] Liu X. J. Qu H. Y. Gou M. X. Guo H. Y. Wang L. Y. Yan X. H. (2020). Application of *Weissella cibaria* X31 or *Weissella confusa* L2 as a starter in low nitrite dry-fermented sausages. Int. J. Food Eng. 16:20190344. doi: 10.1515/ijfe-2019-0344

[ref28] Makarova K. S. Wolf Y. I. Koonin E. V. (2009). Comprehensive comparative-genomic analysis of type 2 toxin-antitoxin systems and related mobile stress response systems in prokaryotes. Biol. Direct 4:19. doi: 10.1186/1745-6150-4-19, PMID: 19493340 PMC2701414

[ref29] Manuse S. Shan Y. Canas-Duarte S. J. Bakshi S. Sun W. S. Mori H.. (2021). Bacterial persisters are a stochastically formed subpopulation of low-energy cells. PLoS Biol. 19:e3001194. doi: 10.1371/journal.pbio.3001194, PMID: 33872303 PMC8084331

[ref30] Muthuramalingam M. White J. C. Bourne C. R. (2016). Toxin-antitoxin modules are pliable switches activated by multiple protease pathways. Toxins. 8, 1–16. doi: 10.3390/toxins8070214, PMID: 27409636 PMC4963847

[ref31] Page R. Peti W. (2016). Toxin-antitoxin systems in bacterial growth arrest and persistence. Nat. Chem. Biol. 12, 208–214. doi: 10.1038/nchembio.2044, PMID: 26991085

[ref32] Shan Y. Gandt A. B. Rowe S. E. Deidsinger J. P. Conlon B. P. Lewis K. (2017). ATP-dependent persister formation in *Escherichia coli*. MBio 8, e02267–e02216. doi: 10.1128/mbio.02267-16, PMID: 28174313 PMC5296605

[ref33] Shen Z. Q. Patil R. D. Sahin O. Wu Z. W. Pu X. Y. Dai L.. (2016). Identification and functional analysis of two toxin-antitoxin systems in *Campylobacter jejuni*. Mol. Microbiol. 101, 909–923. doi: 10.1111/mmi.13431, PMID: 27291507

[ref34] Skjerning R. B. Senissar M. Winther K. S. Gerdes K. Brodersen D. E. (2019). The RES domain toxins of RES-Xre toxin-antitoxin modules induce cell stasis by degrading NAD+. Mol. Microbiol. 111, 221–236. doi: 10.1111/mmi.14150, PMID: 30315706

[ref35] Song Y. J. Luo G. H. Zhu Y. B. Li T. Li C. C. He L. H.. (2021). *Pseudomonas aeruginosa* antitoxin HigA functions as a diverse regulatory factor by recognizing specific pseudopalindromic DNA motifs. Environ. Microbiol. 23, 1541–1558. doi: 10.1111/1462-2920.15365, PMID: 33346387

[ref9002] Song Y. J. Zhang S. P. Ye Z. R. Song Y. Y. Chen L. Tong A. P.. (2022). The novel type II toxin-antitoxin PacTA modulates Pseudomonas aeruginosa iron homeostasis by obstructing the DNA-binding activity of Fur. Nucleic Acids. Res. 50, 10586–10600. 36200834 10.1093/nar/gkac867 PMC9561280

[ref36] Taggar R. Jangra M. Dwivedi A. Bansal K. Patil P. B. Bhattacharyya M. S.. (2021). Bacteriocin isolated from the natural inhabitant of *Allium cepa* against *Staphylococcus aureus*. World. J. Microb. Biot. 37:20. doi: 10.1007/s11274-020-02989-x, PMID: 33427970

[ref37] Urban-Chmiel R. Marek A. Stepien-Pysniak D. Wieczorek K. Dec M. Nowaczek A.. (2022). Antibiotic resistance in bacteria-a review. Antibiotics 11:1079. doi: 10.3390/antibiotics11081079, PMID: 36009947 PMC9404765

[ref38] Wilmaerts D. Bayoumi M. Dewachter L. Knapen W. Mika J. T. Hofkens J.. (2019a). The persistence-inducing toxin HokB forms dynamic pores that cause ATP leakage. MBio 9, e00744–e00718. doi: 10.1128/mbio.00744-18 PMC6094483 30108166

[ref39] Wilmaerts D. Windels E. M. Verstraeten N. Michiels J. (2019b). General mechanisms leading to persister formation and awakening. Trends Genet. 35, 401–411. doi: 10.1016/j.tig.2019.03.007, PMID: 31036343

[ref40] Xiang W. L. Zhang N. D. Lu Y. Zhao Q. H. Xu Q. Rao Y.. (2020). Effect of *Weissella cibaria* co-inoculation on the quality of Sichuan pickle fermented by *Lactobacillus plantarum*. LWT-Food. sci. Technol. 121:108975. doi: 10.1016/j.lwt.2019.108975

[ref41] Xiang W. L. Zhao Q. H. Lu Y. Tang J. Cai T. Rao Y.. (2022). Tetracycline residue alters profile of lactic acid bacterial communities and metabolites of ginger pickle during spontaneous fermentation. Food Res. Int. 155:111109. doi: 10.1016/j.foodres.2022.111109, PMID: 35400400

[ref42] Yu Z. Goodall E. C. A. Henderson I. R. Guo J. (2023). Plasmids can shift bacterial morphological response against antibiotic stress. Adv. Sci. 10:Article 2203260. doi: 10.1002/advs.202203260, PMID: 36424175 PMC9839882

[ref43] Zhang S. Han W. Liu T. Q. Feng C. C. Jiang Q. Zhang B.. (2024). Tetracycline inhibits the nitrogen fixation ability of soybean (*Glycine max* (L.) Merr.) nodules in black soil by altering the root and rhizosphere bacterial communities. Sci. Total Environ. 908:168047. doi: 10.1016/j.scitotenv.2023.168047, PMID: 37918730

[ref44] Zhou X. F. Eckart M. R. Shapiro L. (2021). A bacterial toxin perturbs intracellular amino acid balance to induce persistence. MBio 12:e03020. doi: 10.1128/mbio.03020-20, PMID: 33622732 PMC8545095

[ref45] Zhu H. Y. Xiang W. L. Cai T. Zhang M. Wang H. Y. (2024). PemK's Arg24 is a crucial residue for PemIK toxin–antitoxin system to induce the persistence of *Weissella cibaria* against ciprofloxacin stress. Front. Microbiol. 15:2319. doi: 10.3389/fmicb.2024.1402319, PMID: 38808277 PMC11130411

[ref46] Zorzini V. Buts L. Schrank E. Sterckx Y. G. J. Pespondek M. Engelberg-Kulka H.. (2015). *Escherichia coli* antitoxin MazE as a transcription factor: insights into MazE-DNA binding. Nucleic Acids Res. 43, 1241–1256. doi: 10.1093/nar/gku1352, PMID: 25564525 PMC4333400

