# Supplementary material for: The Bro-Xre toxin-antitoxin modules in Weissella cibaria: inducing persister cells to escape tetracycline stress by disrupting metabolism
Source: Front Microbiol. 2024 Nov 29;15:1505841. doi: 10.3389/fmicb.2024.1505841 (PMC11638225; doi:10.3389/fmicb.2024.1505841)
Supplement: Supplementary file 1 [file Data_Sheet_1.docx]

Supplementary Material

# Supplementary Figures and Tables

For more information on Supplementary Material and for details on the different file types accepted, please see here.

## Supplementary Figures

**Supplementary Figure 1** Homologous alignment of Bro (A) and Xre (B) amino acid sequences. α: α-helix; β: β-sheet. ‘:’ ‘.’ and ‘*’ represent conservation, the degree of conservatism in sequence is ‘*’ ‘:’ ‘.’. Different amino acids with same color represented similar biophysical characteristics (blue: hydrophobic; magenta: polar and negative charge; red: polar and positive charge; green: hydrophilic; glaucous: polar with aromatic side chain; yellow: imino acid; orange: hydrophilic, but non-polar).

**Supplementary Figure 2** Protein homology model of the Bro (A) and Xre (B) of *W. cibaria* 018, and the evaluation of the Bro (C) and Xre (D) protein.

**Supplementary Figure 3** Effects of Bro on cell membrane potential, ATP content and related metabolism in *E. coli* BL21. A: The change of cell membrane potential in *E. coli* BL21*.* The samples were obtained by treated with 2.0 g/L arabinose (indued the Bro protein). B: The change of ATP content in *E. coli* BL21*.* The samples were obtained by treated with 2.0 g/L arabinose (indued the Bro protein). C: The effect on gene expression of energy metabolism in *E. coli* BL21 by induced the Bro protein. D: The effect on gene expression of amino acid synthesis in *E. coli* BL21 by induced the Bro protein. E: The effect on gene expression of nucleotide synthesis in *E. coli* BL21 by induced the Bro protein.

**Supplementary Figure 4** The effect of Bro on the morphology and the formation of persister cells of recombinant *E. coli* BL21. A: Effect of Bro protein on the cell length. B: The frequency of persister cells of *E. coli* BL21 and it was performed by biphasic extinction curve. C: The effect of Bro of *E. coli* BL21 on the cell morphology.

## Supplementary Tables

**Supplementary Table** **1**. Interface summary of Xre_(1)_-Xre_(2)_

**Supplementary Table** **2**. Hydrogen bound of Xre_(1)_-Xre_(2)_

**Supplementary Table** **3**. Salt bridges of Xre_(1)_-Xre_(2)_

**Supplementary Table** **4**. Xre_(1)_-bound promoter interface residue pairs

**Supplementary Table** **5**. Xre_(2)_-bound promoter interface residue pairs

**Supplementary Table** **6**. Interface summary of Bro_(1)_-Bro_(2)_

**Supplementary Table** **7**. Hydrogen bound of Bro_(1)_-Bro_(2)_

**Supplementary Table** **8**. Interface summary of Xre_(1)_-Bro_(1)_

**Supplementary Table** **9**. Hydrogen bound of Xre_(1)_-Bro_(1)_

**Supplementary Table** **10**. Salt bridges of Xre_(1)_-Bro_(1)_

**Supplementary Table** **11**. Interface summary of Xre_(2)_-Bro_(1)_

**Supplementary Table** **12**. Hydrogen bound of Xre_(2)_-Bro_(1)_

**Supplementary Table** **13**. Salt bridges of Xre_(2)_-Bro_(1)_


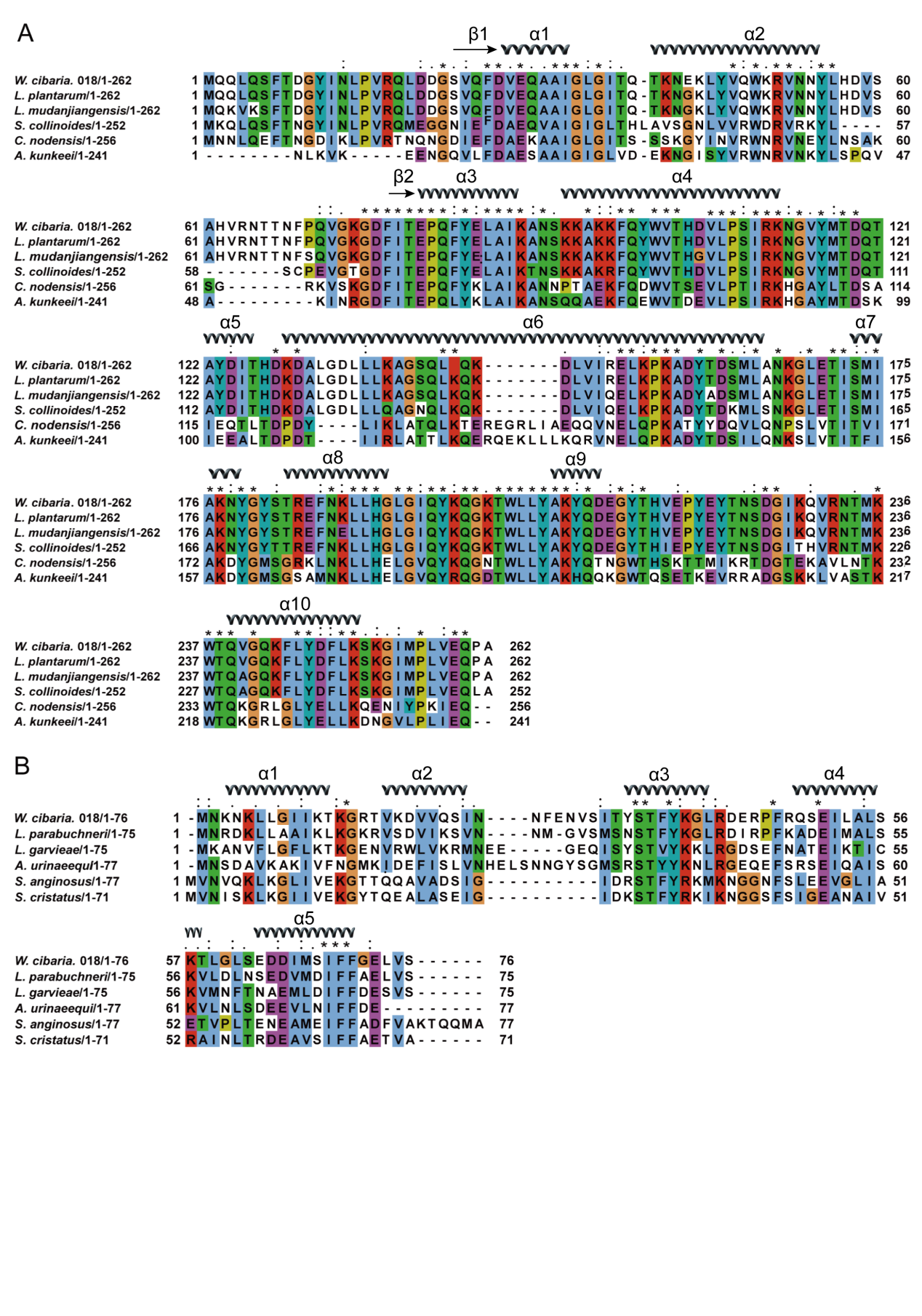


**Supplementary Figure 1** Homologous alignment of Bro (A) and Xre (B) amino acid sequences. α: α-helix; β: β-sheet. ‘:’ ‘.’ and ‘*’ represent conservation, the degree of conservatism in sequence is ‘*’ ‘:’ ‘.’. Different amino acids with same color represented similar biophysical characteristics (blue: hydrophobic; magenta: polar and negative charge; red: polar and positive charge; green: hydrophilic; glaucous: polar with aromatic side chain; yellow: imino acid; orange: hydrophilic, but non-polar).


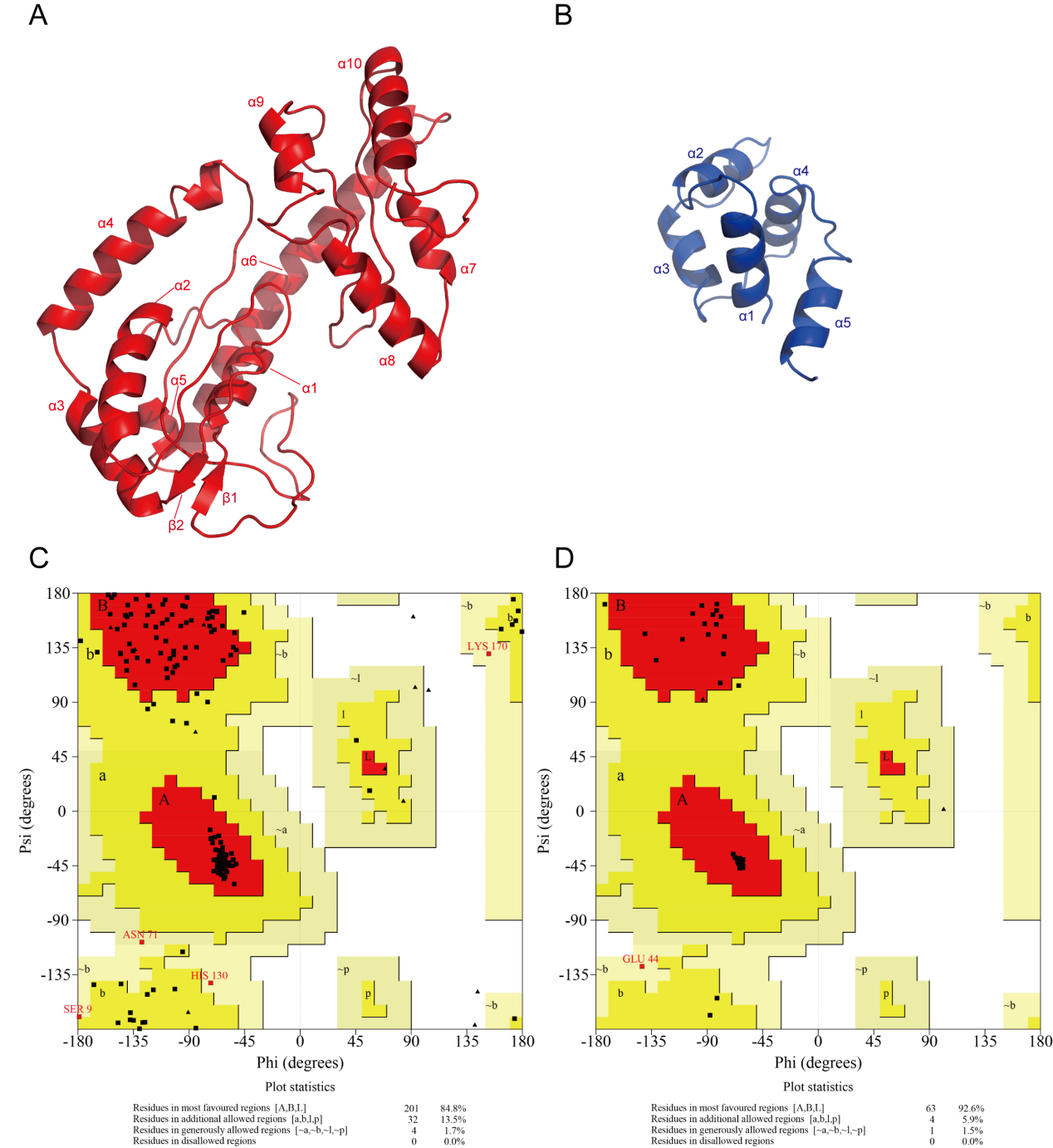
 **Supplementary Figure 2** Protein homology model of the Bro (A) and Xre (B) of *W. cibaria* 018, and the evaluation of the Bro (C) and Xre (D) protein.


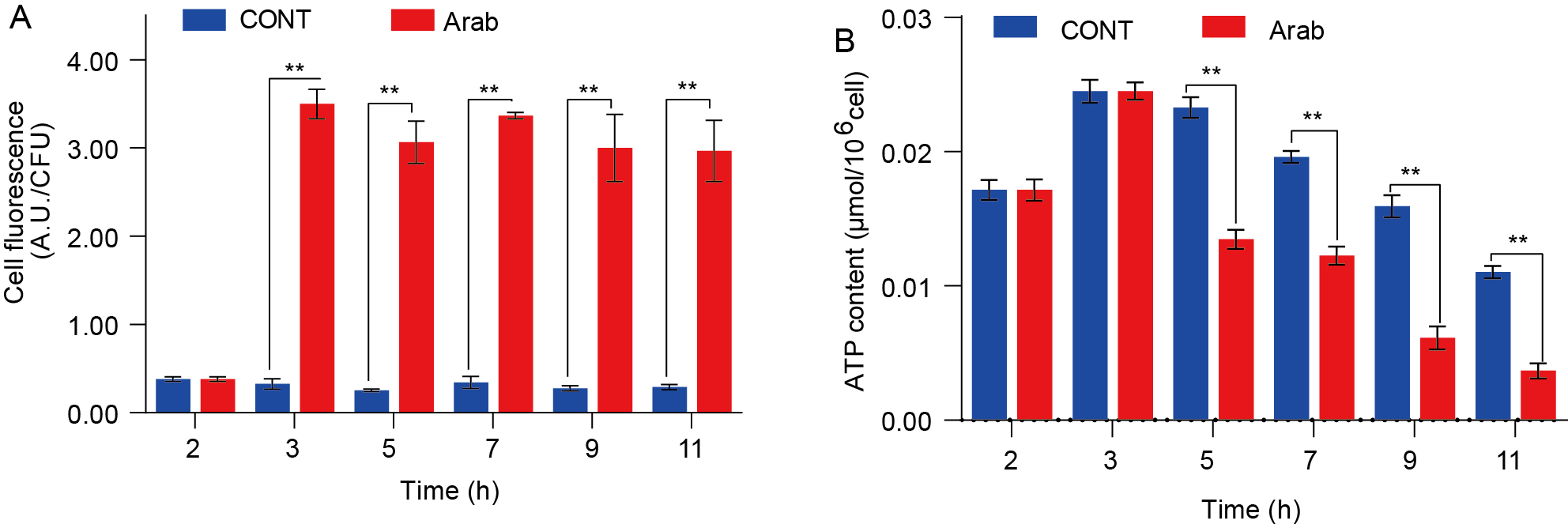


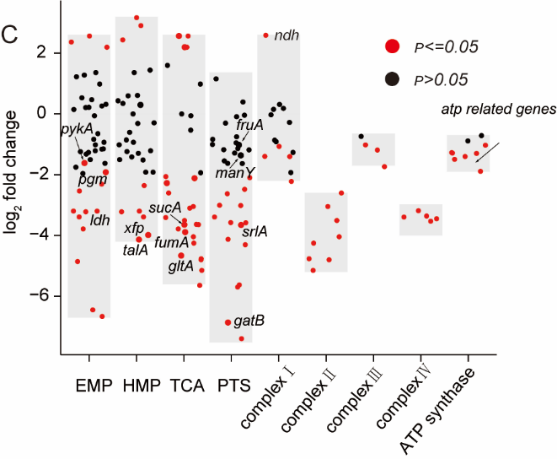

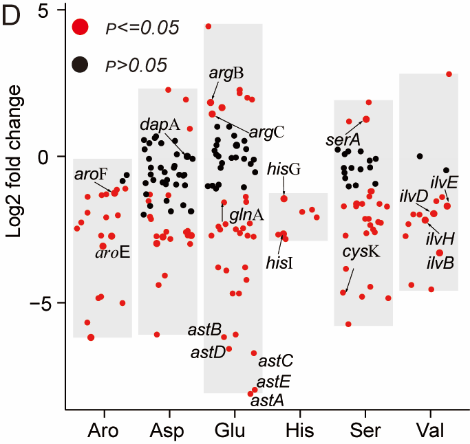

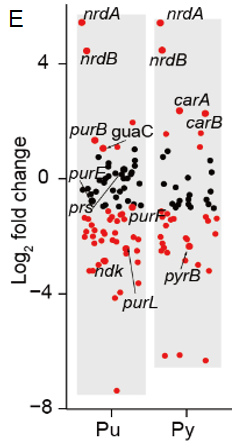


**Supplementary Figure 3** Effects of Bro on cell membrane potential, ATP content and related metabolism in *E. coli* BL21. A: The change of cell membrane potential in *E. coli* BL21*.* The samples were obtained by treated with 2.0 g/L arabinose (indued the Bro protein). B: The change of ATP content in *E. coli* BL21*.* The samples were obtained by treated with 2.0 g/L arabinose (indued the Bro protein). C: The effect on gene expression of energy metabolism in *E. coli* BL21 by induced the Bro protein. D: The effect on gene expression of amino acid synthesis in *E. coli* BL21 by induced the Bro protein. E: The effect on gene expression of nucleotide synthesis in *E. coli* BL21 by induced the Bro protein.


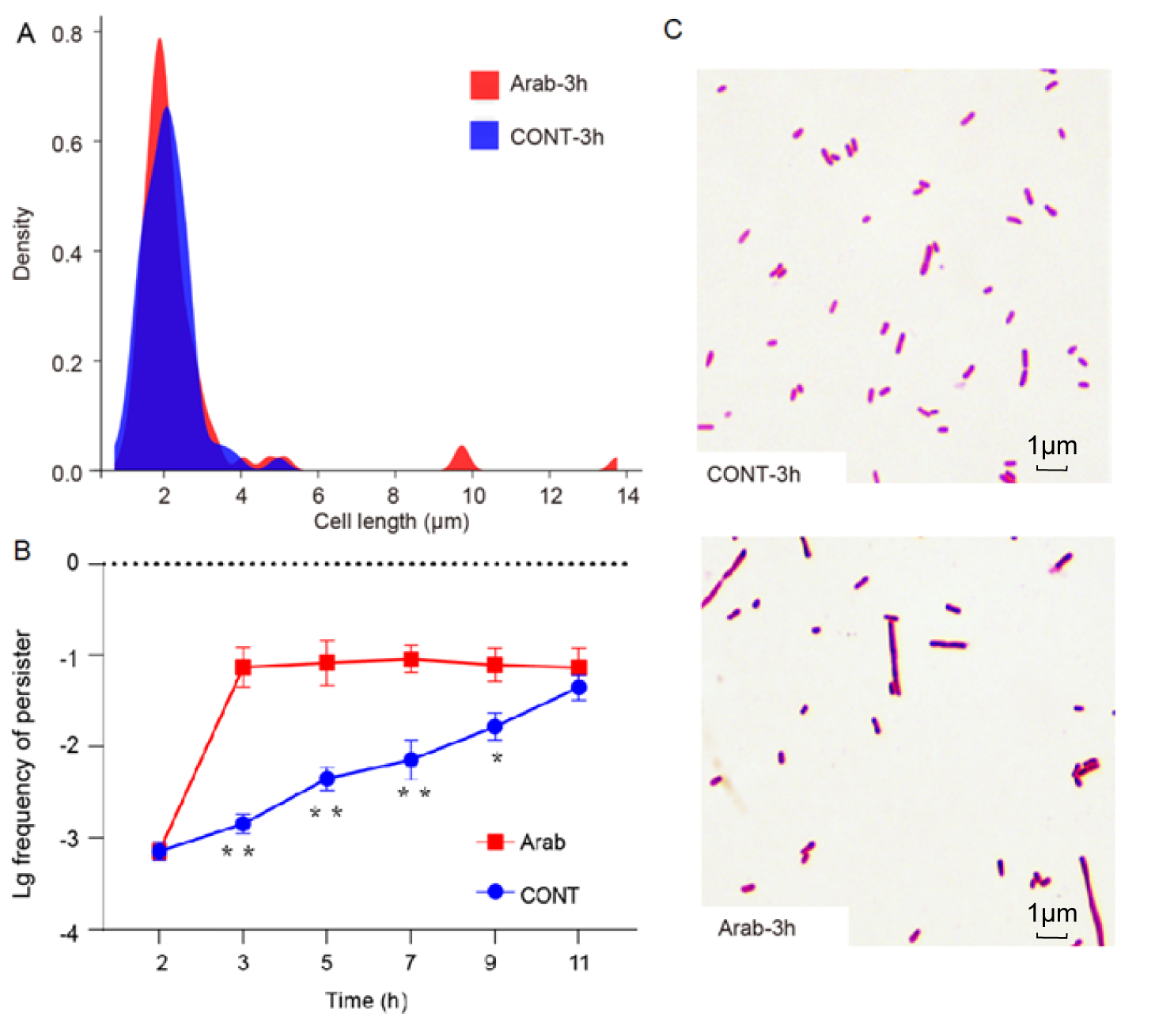


**Supplementary Figure 4**. The effect of Bro on the morphology and the formation of persister cells of recombinant *E. coli* BL21. A: Effect of Bro protein on the cell length. B: The frequency of persister cells of *E. coli* BL21 and it was performed by biphasic extinction curve. C: The effect of Bro of *E. coli* BL21 on the cell morphology.

**Supplementary Table** **1**. Interface summary of Xre_(1)_-Xre_(2)_

| Interface summary | | Xre_(1)_ | | Xre_(2)_ | |
| --- | --- | --- | --- | --- | --- |
| List of Interfaces | Interface area, Å^2^ | 973.5 | | | |
|  | Δ^i^G, kcal/mol | -10.5 | | | |
|  | Δ^i^G, P-value | 0.243 | | | |
| Number of atoms | interface | 98 | 16.1% | 96 | 15.8% |
|  | surface | 412 | 67.8% | 413 | 67.9% |
|  | total | 608 | 100.0% | 608 | 100.0% |
| Number of residues | interface | 28 | 36.8% | 27 | 35.5% |
|  | surface | 76 | 100.0% | 76 | 100.0% |
|  | total | 76 | 100.0% | 76 | 100.0% |
| Solvent-accessible area, Å | interface | 995.3 | 18.4% | 951.8 | 17.6% |
|  | total | 5414.1 | 100.0% | 5418.3 | 100.0% |
| Solvation energy, kcal/mol | isolated structure | -63.1 | 100.0% | -63.0 | 100.0% |
|  | Gain on complex formation | -7.8 | 12.4% | -2.7 | 4.3% |
|  | average gain | -3.2 | 5.1% | -3.2 | 5.0% |
|  | P-value | 0.105 | | 0.562 | |

**Supplementary Table** **2**. Hydrogen bound of Xre_(1)_-Xre_(2)_

| Hydrogen bound | | Xre_(1)_ | Dist. [Å] | Xre_(2)_ |
| --- | --- | --- | --- | --- |
| 1 | SER 68 | | 3.15 | TYR 38 |
| 2 | GLN 49 | | 2.20 | LYS 5 |
| 3 | PHE 47 | | 3.41 | THR 12 |
| 4 | ASP 64 | | 2.93 | ARG 42 |

**Supplementary Table** **3**. Salt bridges of Xre_(1)_-Xre_(2)_

| Salt bridges | Xre_(1)_ | Dist. [Å] | Xre_(2)_ |
| --- | --- | --- | --- |
| 1 | ASP 64 | 3.84 | ARG 42 |
| 2 | ASP 64 | 2.93 | ARG 42 |

**Supplementary Table** **4**. Xre_(1)_-bound promoter interface residue pairs

| Residue pairs | Promoter(5’-3’) | Dist. [Å] | Xre_(1)_ |
| --- | --- | --- | --- |
| 1 | DT1 | 3.422 | LEU53 |
| 2 | DT1 | 3.607 | LEU61 |
| 3 | DT1 | 2.650 | SER62 |
| 4 | DT1 | 1.807 | GLU63 |
| 5 | DT2 | 3.209 | SER62 |
| 6 | DT2 | 3.272 | ASP64 |
| 7 | DG54 | 2.276 | SER68 |
| 8 | DG54 | 3.207 | PHE71 |
| 9 | DG54 | 3.010 | GLY72 |
| 10 | DT55 | 3.505 | ASP64 |
| 11 | DT55 | 2.879 | ASP65 |
| 12 | DT55 | 3.016 | SER68 |
| 13 | DT55 | 2.620 | ILE69 |
| 14 | DC56 | 3.981 | ASP65 |
| 15 | DA57 | 2.933 | SER62 |

**Supplementary Table** **5**. Xre_(2)_-bound promoter interface residue pairs

| Residue pairs | Promoter(5’-3’) | Dist. [Å] | Xre_(2)_ |
| --- | --- | --- | --- |
| 1 | DT1 | 3.905 | GLU73 |
| 2 | DT2 | 2.127 | MET1 |
| 3 | DT2 | 3.406 | LYS3 |
| 4 | DT2 | 3.772 | ASN4 |
| 5 | DT2 | 3.744 | ASP43 |
| 6 | DT2 | 3.762 | LEU74 |
| 7 | DG3 | 3.886 | ARG42 |
| 8 | DG3 | 2.441 | ASP43 |
| 9 | DA4 | 2.311 | ARG42 |
| 10 | DA52 | 2.714 | SER35 |
| 11 | DT53 | 2.989 | TYR34 |
| 12 | DT53 | 3.484 | SER35 |
| 13 | DT53 | 2.392 | TYR38 |
| 14 | DT53 | 3.565 | LYS39 |
| 15 | DG54 | 2.693 | TYR38 |
| 16 | DG54 | 2.024 | ARG42 |
| 17 | DT55 | 2.562 | ARG42 |

**Supplementary Table 6**. Interface summary of Bro_(1)_-Bro_(2)_

| Interface summary | | Bro_(1)_ | | Bro_(2)_ | |
| --- | --- | --- | --- | --- | --- |
| List of Interfaces | Interface area, Å^2^ | 616.8 | | | |
|  | Δ^i^G, kcal/mol | -0.1 | | | |
|  | Δ^i^G, P-value | 0.657 | | | |
| Number of atoms | interface | 172 | 8.00% | 163 | 7.60% |
|  | surface | 1438 | 66.70% | 1425 | 66.10% |
|  | total | 2155 | 100.00% | 2155 | 100.00% |
| Number of residues | interface | 48 | 18.10% | 45 | 17.00% |
|  | surface | 262 | 98.90% | 263 | 99.20% |
|  | total | 265 | 100.00% | 265 | 100.00% |
| Solvent-accessible area, Å | interface | 1522.1 | 9.20% | 1489.4 | 9.00% |
|  | total | 16509.3 | 100.00% | 16501 | 100.00% |
| Solvation energy, kcal/mol | isolated structure | -202.4 | 100.00% | -202.3 | 100.00% |
|  | Gain on complex formation | -8.9 | 4.40% | -6.3 | 3.10% |
|  | average gain | -7.3 | 3.60% | -7 | 3.50% |
|  | P-value | 0.368 | | 0.568 | |

**Supplementary Table** **7**. Hydrogen bound of Bro_(1)_-Bro_(2)_

| Hydrogen bound | | Bro_(1)_ | Dist. [Å] | Bro_(2)_ |
| --- | --- | --- | --- | --- |
| 1 | LYS 77 | | 3.69 | GLN 8 |
| 2 | THR 186 | | 3.77 | THR 11 |
| 3 | THR 186 | | 2.78 | ASP 12 |
| 4 | ARG 20 | | 2.93 | ASN 16 |
| 5 | ARG 20 | | 3.03 | LEU 17 |
| 6 | LYS 77 | | 3.79 | GLN 21 |
| 7 | ASP 23 | | 3.80 | GLY 76 |
| 8 | ASP 12 | | 2.78 | TYR 184 |
| 9 | TYR 184 | | 3.69 | GLY 13 |
| 10 | GLN 8 | | 2.10 | GLN 33 |
| 11 | GLN 21 | | 2.02 | GLN 33 |
| 12 | GLN 8 | | 3.83 | GLY 76 |
| 13 | PHE 10 | | 3.67 | TYR 184 |

**Supplementary Table** **8**. Interface summary of Xre_(1)_-Bro_(1)_

| Interface summary | | Xre_(1)_ | | Bro_(1)_ | |
| --- | --- | --- | --- | --- | --- |
| List of Interfaces | Interface area, Å^2^ | 1119.8 | | | |
|  | Δ^i^G, kcal/mol | -7.3 | | | |
|  | Δ^i^G, P-value | 0.540 | | | |
| Number of atoms | interface | 107 | 17.6% | 110 | 5.1% |
|  | surface | 412 | 67.8% | 1438 | 66.7% |
|  | total | 608 | 100.0% | 2155 | 100.0% |
| Number of residues | interface | 29 | 38.2% | 32 | 12.1% |
|  | surface | 76 | 100.0% | 262 | 98.9% |
|  | total | 76 | 100.0% | 265 | 100.0% |
| Solvent-accessible area, Å | interface | 1136.5 | 21.0% | 1103.0 | 6.7% |
|  | total | 5414.1 | 100.0% | 16509.3 | 100.0% |
| Solvation energy, kcal/mol | isolated structure | -63.1 | 100.0% | -202.4 | 100.0% |
|  | Gain on complex formation | -2.0 | 3.2% | -5.2 | 2.6% |
|  | average gain | -3.5 | 5.6% | -4.7 | 2.3% |
|  | P-value | 0.649 | | 0.449 | |

**Supplementary Table** **9**. Hydrogen bound of Xre_(1)_-Bro_(1)_

| Hydrogen bound | | Xre_(1)_ | Dist. [Å] | Bro_(1)_ |
| --- | --- | --- | --- | --- |
| 1 | ILE 9 | | 2.54 | LYS 115 |
| 2 | GLU 63 | | 2.80 | TYR 49 |
| 3 | GLU 73 | | 2.86 | TYR 209 |
| 4 | GLU 73 | | 3.50 | GLN 213 |
| 5 | SER 76 | | 3.40 | ALA 210 |

**Supplementary Table** **10**. Salt bridges of Xre_(1)_-Bro_(1)_

| Salt bridges | Xre_(1)_ | Dist. [Å] | Bro_(1)_ |
| --- | --- | --- | --- |
| 1 | ARG 15 | 3.50 | ASP 108 |
| 2 | ARG 15 | 3.60 | ASP 108 |
| 3 | ARG 15 | 3.86 | ASP 108 |
| 4 | SER 76 | 3.84 | LYS 255 |

**Supplementary Table** **11**. Interface summary of Xre_(2)_-Bro_(1)_

| Interface summary | | Xre_(2)_ | | Bro_(1)_ | |
| --- | --- | --- | --- | --- | --- |
| List of Interfaces | Interface area, Å^2^ | 949.1 | | | |
|  | Δ^i^G, kcal/mol | -4.0 | | | |
|  | Δ^i^G, P-value | 0.685 | | | |
| Number of atoms | interface | 87 | 14.3% | 106 | 4.9% |
|  | surface | 413 | 679% | 1438 | 66.7% |
|  | total | 608 | 100.0% | 2155 | 100.0% |
| Number of residues | interface | 23 | 30.3% | 26 | 9.8% |
|  | surface | 76 | 100.0% | 262 | 98.9% |
|  | total | 76 | 100.0% | 265 | 100.0% |
| Solvent-accessible area, Å | interface | 1014.7 | 18.7% | 883.4 | 5.4% |
|  | total | 5418.3 | 100.0% | 16509.3 | 100.0% |
| Solvation energy, kcal/mol | isolated structure | -63.0 | 100.0% | -202.4 | 100.0% |
|  | Gain on complex formation | -1.8 | 2.9% | -2.2 | 1.1% |
|  | average gain | -2.9 | 4.5% | -4.5 | 2.2% |
|  | P-value | 0.620 | | 0.757 | |

**Supplementary Table** **12**. Hydrogen bound of Xre_(2)_-Bro_(1)_

| Hydrogen bound | | Xre_(2)_ | Dist. [Å] | Bro_(1)_ |
| --- | --- | --- | --- | --- |
| 1 | LYS 3 | | 2.45 | THR 69 |
| 2 | SER 35 | | 3.40 | HIS 194 |
| 3 | TYR 34 | | 3.50 | THR 205 |
| 4 | ARG 42 | | 2.70 | ASN 71 |
| 5 | ASP 43 | | 2.60 | ASN 68 |
| 6 | SER 76 | | 3.00 | ASN 57 |

**Supplementary Table** **13**. Salt bridges of Xre_(2)_-Bro_(1)_

| Salt bridges | Xre_(2)_ | Dist. [Å] | Bro_(1)_ |
| --- | --- | --- | --- |
| 1 | SER 76 | 3.80 | ARG 54 |
